# Selenium Speciation Analysis Reveals Improved Antioxidant Status in Finisher Pigs Fed l-Selenomethionine, Alone or Combined with Sodium Selenite, and Vitamin E

**DOI:** 10.1007/s12011-022-03516-9

**Published:** 2022-12-29

**Authors:** Estela Reinoso-Maset, Michaela Falk, Aksel Bernhoft, Cecilie Ersdal, Tore Framstad, Herbert Fuhrmann, Brit Salbu, Marianne Oropeza-Moe

**Affiliations:** 1 Centre for Environmental Radioactivity CoE, Faculty of Environmental Sciences and Natural Resource Management, Norwegian University of Life Sciences, Elizabeth Stephansens vei 31, 1433 Aas, Norway; 2 Norwegian Veterinary Institute, Svebastadveien 112, 4325 Sandnes, Norway; 3 Department of Production Animal Clinical Sciences (PRODMED), Faculty of Veterinary Medicine, Norwegian University of Life Sciences, Svebastadveien 112, 4325 Sandnes, Norway; 4 Norwegian Veterinary Institute, Elizabeth Stephansens vei 1, 1433 Aas, Norway; 5 Department of Production Animal Clinical Sciences (PRODMED), Faculty of Veterinary Medicine, Norwegian University of Life Sciences, Elizabeth Stephansens vei 15, 1433 Aas, Norway; 6 Institute of Physiological Chemistry, Faculty of Veterinary Medicine, University of Leipzig, 04103 Leipzig, Germany

**Keywords:** Organic selenium, Vitamin E, Selenospecies, GPx3, Finisher pigs

## Abstract

Conditions associated with selenium (Se) and/or vitamin E (VitE) deficiency are still being reported in high-yielding pigs fed the recommended amounts. Here, the dietary effects of Se source (sodium selenite, NaSe, 0.40 or 0.65 mg Se/kg; l-selenomethionine, SeMet, 0.19 or 0.44 mg Se/kg; a NaSe-SeMet mixture, SeMix, 0.44–0.46 mg Se/kg) and VitE concentration (27, 50–53 or 101 mg/kg) on the antioxidant status of finisher pigs were compared with those in pigs fed non-Se-supplemented diets (0.08–0.09 mg Se/kg). Compared to NaSe-enriched diets, SeMet-supplemented diets resulted in significantly (*p* < 0.0018) higher plasma concentrations of total Se (14–27%) and selenospecies (GPx3, SelP, SeAlb; 7–83%), significantly increased the total Se accumulation in skeletal muscles, myocardium, liver and brain (10–650%), and enhanced the VitE levels in plasma (15–74%) and tissues (8–33%) by the end of the 80-day trial, proving better Se distribution and retention in pigs fed organic Se. Injecting lipopolysaccharide (LPS) intravenously half-way into the trial provoked a pyrogenic response in the pigs followed by a rapid increase of inorganic Se after 5–12 h, a drastic drop of SeMet levels between 12 and 24 h that recovered by 48 h, and a small increase of SeCys by 24–48 h, together with a gradual rise of GPx3, SelP and SeAlb in plasma up to 48 h. These changes in Se speciation in plasma were particularly significant (0.0024 > *p* > 0.00007) in pigs receiving SeMet- (0.44 mg Se/kg, above EU-legislated limits) or SeMix-supplemented (SeMet and NaSe both at 0.2 mg Se/kg, within EU-legislated limits) diets, which demonstrates Se metabolism upregulation to counteract the LPS-induced oxidative stress and a strengthened antioxidant capacity in these pigs. Overall, a Se source combination (without exceeding EU-legislated limits) and sufficient VitE supplementation (≥ 50 mg/kg) improved the pigs’ antioxidant status, while doubling the allowed dietary organic Se increased the Se in tissues up to sixfold without compromising the animal’s health due to toxicity. This study renders valuable results for revising the current dietary SeMet limits in swine rations.

**Graphical Abstract:**

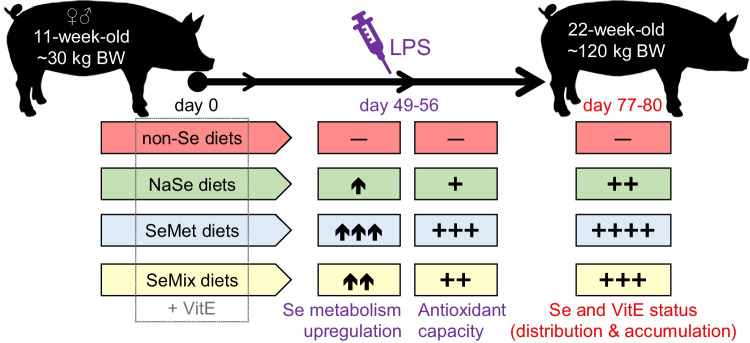

**Supplementary Information:**

The online version contains supplementary material available at 10.1007/s12011-022-03516-9.

## Introduction

Breeding programs in pig production have improved financial profit by focusing on beneficial traits like weight gain, leanness and feed efficiency [1]; however, questions have been raised whether high-yielding pigs receive sufficient selenium (Se) in their feed [2, 3] and if not, whether there is resource allocation [4], meaning that when nutritional resources are scarce there is a trade-off between different biological processes, e.g. favouring growth over immunity, and consequently pigs might become more susceptible to oxidative assaults. For example, the Norwegian hybrid finisher lines have had an increase of average lean meat between 2007 and 2020 (from 56.5 to 60.6%), while the average net feed units per kg weight gain have been reduced in both weaned (from 1.81 to 1.70) and grower-finisher (from 2.74 to 2.68) pigs [5]. This means that, since the dietary upper limits of supplementation have remained unchanged during the same period, there has been a net reduction in available nutrients, such as Se, and in turn the animal’s antioxidant status is likely being compromised [6].

Selenium and vitamin E (VitE) are essential nutrients with antioxidant functions that can prevent oxidative damage to biological membranes and enhance immune responses [7, 8]. Selenium, as the selenized amino acid selenocysteine (SeCys) residue, is an essential structural component in selenoproteins (e.g. the Se transport and storage protein selenoprotein P (SelP) and the antioxidant enzyme extracellular glutathione peroxidase (GPx3)) that play a key role in protecting cells against oxidative stress by neutralizing and degrading reactive oxygen species (ROS) [9, 10]. Free selenomethionine (SeMet) can also serve as a ROS-scavenger [9] and, as a constituent of proteins such as selenoalbumin (SeAlb; formed by non-specific incorporation of SeMet in lieu of methionine in albumin), contribute to maintain enhanced Se status for longer periods [11]. On the other hand, VitE is the major lipid-soluble antioxidant present in all cellular membranes that can directly scavenge a variety of free radicals and ROS by limiting peroxidation [7] and is also known to interact synergistically with Se in the antioxidant defence system [7, 8, 12]. Therefore, deficiency of these essential nutrients can disturb the critical balance between free radical generation and antioxidant defences, leading to oxidative damage that may cause degeneration of muscle fibres [13] associated with severe heart and muscular conditions. In high-yielding pigs, Se and VitE deficiency is known to cause the conditions mulberry heart disease (MHD) and nutritional muscular dystrophy (NMD) [3, 6, 14], and signs of these are still being reported [15–18] despite that the dietary recommendations are met.

The fortification of animal diets with Se and VitE has been proven to improve growth and disease resistance in pigs [19–21]; yet, the uptake, accumulation and metabolic function will depend on the supplementation source and status of the animals [22, 23]. The dietary Se requirement can be attained by enriching the feed with Se in an inorganic form, such as sodium selenite (Na_2_SeO_3_), or with organic compounds, such as SeMet (2-amino-4-(methylselanyl)butanoic acid), but the total concentrations in the feed must be within legislated values. For countries attained to the European Commission regulations, the levels are restricted to 0.5 mg Se/kg in the complete feed, i.e. contribution from raw materials plus any supplement, though only up to 0.2 mg Se/kg can be from an organic source [24, 25]. Since SeMet is the only Se form that allows building long-term Se body reserves [26–29], i.e. what is not directly metabolized into selenoproteins in the liver can be incorporated into tissues with high rates of body protein synthesis (e.g. skeletal muscles [30]) and retained in brain proteins and haemoglobin [11, 26], the uptake, retention and bioavailability of organic Se are expected to be higher and the whole-body half-life of Se to be longer than those resulting from inorganic Se dietary supplementation [31–36]. Moreover, the addition of VitE to Se-enriched diets at a sufficient level should further improve the status in the pigs [7, 37]. Nonetheless, even if the requirements are based on the interrelationship between both nutrients [38], dietary studies with pigs often investigate the effect of Se source and VitE separately.

The aim of the present work was therefore to study the effects of feed enriched with selenite, SeMet or a mixture of these Se sources and in combination with VitE on the antioxidant status of high-yielding, finisher pigs and their response capacity towards oxidative stress. These effects were determined by evaluating the pigs’ health, nutrient uptake and accumulation in plasma and tissues, and Se speciation in plasma during growth over a 80-day dietary trial and during 48 h after a lipopolysaccharide (LPS) injection half-way into the trial.

## Materials and Methods

### Study Design

This animal trial was conducted at the research unit of Felleskjøpet Rogaland and Agder (Klepp, Norway). The Norwegian Food Safety Authority approved the trial, which complied with the current European and Norwegian Animal Welfare Act (LOV-2009–06-19–97) and the Norwegian regulations on swine husbandry (FOR-2003–02-18–175). At the end of the trial, the pigs were slaughtered by standard procedures at a local abattoir (Nortura Forus).

#### Animals and Housing

The 80-day feeding trial included 128 finisher pigs (*Landrace*/*Yorkshire* (sow) x *Duroc*/*Duroc* (boar) hybrids; 1:1 gender ratio; 11-week-old; 29.1 ± 3.6 kg initial average body weight (BW)) that had been vaccinated against *Porcine circovirus 2* (Circovac, Merial, France) at 3 weeks of age and against *Actinobacillus pleuropneumoniae* (Porcilis APP Vet., MSD Animal health, Netherlands) at 6 and 10 weeks of age. Pigs were randomly distributed into 8 groups, i.e. 16 pigs were assigned per treatment. Pigs receiving the same treatment were allocated into two pens of 7 and 9 pigs, which were 8.10 and 10.79 m^2^ in size with 2.78 and 3.15 m^2^ of slatted floor, respectively, and natural wood shavings and straw as bedding material. The initial room temperature of 23.5 °C was reduced daily by ~ 0.1 °C, reaching 14.5 °C by the end of the trial, and the light:dark periods were set to 16:8 h.

Each pen was equipped with a FIRE® system (Feed Intake Recording Equipment, Osborne Ltd, USA) and transponder ear tags with unique ID numbers were attached to the right ear of each pig at arrival to the research unit. The FIRE® systems were verified by individually weighing the pigs prior to start and comparing to the weight obtained from the corresponding pen’s feeder station [39]. These electronic automatic feeders together with the ear tags allowed to individually monitor the pigs and automatically record their BW (in kg), feed intake (in kg) and number of visits for every day of the trial. Moreover, the FIRE stations provided *ad libitum* access to the feed as well as water throughout the trial. This experimental setup hence provided an acceptable tool for unbiasedly collecting accurate data of individual animals housed in a group environment; and, due to the unrestricted feeding regime and identical group and pen sizes between treatments, it did not introduce an external time factor or group size effect that could interfere with the pigs’ feeding pattern and natural growth, i.e. in this study the individual animal was considered the experimental unit [40].

#### Dietary Study

Prior to entering the feeding trial, the 128 pigs had been fed a standard compound starter feed containing 18% crude protein, 1.2% lysine and 5.3% crude fat, supplemented with 150 mg VitE/kg and 0.4 mg Se/kg as sodium selenite among other common vitamins and trace minerals for pig feed [31]. During the 80-day dietary trial, each group of pigs (i.e. 16 from both the 7- and 9-pig pens) were provided with feeds containing different Se sources and concentrations as well as different VitE concentrations, which were chosen based on the EU-legislation upper limits of 0.5 mg/kg in the complete feed and 0.2 mg/kg as organic Se supplement [24, 25] and the no upper limit for VitE. The basal diet feed was produced with raw materials of Norwegian origin and thus contained ~ 0.05 mg Se/kg feed. In terms of Se source and concentration, the 8 diet treatments were as follows:

- Selenium deficient, i.e. the non-Se-supplemented basal feed (Control-1 and Control-2 diet groups);
- Basal feed supplemented with inorganic Se in the form of sodium selenite (NaSe; Mikromin Selen 300 FK, Vilomix, Norway) up to 0.40 mg Se/kg (NaSe-0.40 diet group) and 0.65 mg Se/kg (NaSe-0.65 diet group), which were ~ 20% lower and ~ 30% higher than the EU-limit for total Se in complete feed;
- Basal feed supplemented with organic Se in the form of l-selenomethionine (SeMet; Excential Selenium 4000, Orffa, Netherlands) up to 0.19 mg Se/kg (SeMet-0.19 diet group) and 0.44 mg Se/kg (SeMet-0.44 diet group), which were similar and ~ 55% higher than the allowed EU-limit for supplemented organic Se but still under the Se limit for complete feed; or,
- Basal feed supplemented with sodium selenite and SeMet mixed at a mass ratio that provided equimolar Se up to a total Se concentration of 0.44–0.46 mg/kg, which was within the allowed EU-limits for total Se and supplemented organic Se (SeMix-1 and SeMix-2 diet groups).

All diet feeds were also enriched with VitE (alpha-tocopherol; Premix, Vilomix, Norway) up to a total concentration of ca. 50 mg/kg of feed, except for Control-1 and SeMix-2 feeds that were supplemented up to ca. 25 and 100 mg VitE/kg, respectively. Other main dietary constituents were added in equal amounts to all diet feeds, with ca. 15% and 5% of protein and fat, respectively. The composition of the 8 diets was determined in feed sub-samples prior to the trial. Details of nutrients, components, additives and measured concentrations of enriched components in the feed can be found in Table 1.

**Table 1:** Nutrient composition (as % of feed) and dietary components (as % of dry matter) of the basal feed, and the enriched components (Se and VitE; as added in % of dry matter and as measured in mg/kg of feed) of the 8 diet treatments used in the 80-day feeding trial with finisher pigs

*Nutrient* ^1a^	*% of feed*	
Water	11.1 (*0.8*)	
Protein	14.9 (*0.2*)	
Fat	5.6 (*0.2*)	
Ash	4.7 (*0.2*)	
Fibre	4.9 (*0.3*)	
*Dietary component* ^1a^	*% of dry matter*	
Wheat	33.3 (*0.3*)	
Barley	20.0	
Oats	13.4 (*0.7*)	
Soy meal	9.1 (*0.1*)	
Rapeseed cake meal	8.0	
Wheat bran	6.7 (*0.6*)	
Beans	4.0	
Animal fat	2.0	
Feed lime	1.12 (*0.01*)	
Sugar cane molasses	0.50	
Feed salt	0.46	
L-lysine	0.39	
Mono-Ca-phosphate	0.21	
L-threonine	0.18	
Microminerals Pig^2a^	0.16	
DL-methionine	0.12	
Seal4Feed	0.10	
Amasil NA	0.10	
Vitamin ADKB mix^2b^	0.06	
Myco CURB	0.04	
Vitamin A premix	0.03	
PHYZYME XP 5000L	0.014	
L-valine	0.010	
L-tryptophan	0.010	
*Enriched component* ^1a^	*% of dry matter*	
Vitamin E Premix	0.0027, 0.007, 0.0176	
Mikromin Selen 300 FK^3a^	0.07, 0.2, 0.13	
Excential Selenium 4000^3b^	0.012, 0.025	
	*mg/kg of feed*	
*Diet group*	Alpha-tocopherol^1b^	Selenium^1c^
*Control-1*	27.5 *(1.3)*	0.077 (*0.003*)
*Control-2*	50.6 (*0.4*)	0.089 (*0.004*)
*NaSe-0.40*	53.7 (*1.0*)	0.40 (*0.05*)^3a^
*NaSe-0.65*	50.8 (*2.5*)	0.65 (*0.04*)^3a^
*SeMet-0.19*	53.8 (*0.8*)	0.19 (*0.04*)^3b^
*SeMet-0.44*	51.7 (*2.4*)	0.44 (*0.02*)^3b^
*SeMix-1*	53.6 (*0.3*)	0.46 (*0.03*)^3c^
*SeMix-2*	101.1 (*3.3*)	0.44 (*0.03*)^3c^

^1^Analysis methods: ^1a^Dir. 152/2009/EU (average % (standard deviation (SD)), *n* = 8, except for enriched components); ^1b^RP-HPLC with UV-detection (average (SD), *n* = 2); ^1c^ICP-MS (average (SD), *n* = 3)

^2^Additives composition: ^2a^96 mg/kg Fe and 20.8 mg/kg Cu as sulphate salts, 48 mg/kg Mn and 96 mg/kg Zn as oxide salts, 0.48 mg/kg I; ^2b^5700 IU vitamin A, 1200 IU vitamin D, 100 mg/kg vitamin E, 3.72 mg/kg vitamin K, 2.4 mg/kg vitamin B1, 4.5 mg/kg vitamin B2, 12.0 mg/kg vitamin B5, 7.2 mg/kg vitamin B6, 0.012 mg/kg vitamin B12, 1.8 mg/kg folic acid, 0.24 mg/kg biotin

^3^Selenium sources: ^3a^inorganic Se as Na_2_SeO_3_; ^3b^organic Se as L-selenomethionine; ^3c^Se molar ratio of 1.09 for Na_2_SeO_3_:L-selenomethionine

#### LPS Challenge Study

Between 49 and 56 days into the trial (one diet group per day), all the individuals from the 9-pig pens received 2 µg LPS/kg of BW (*Escherichia coli* O111:B4, L4391, lot: 014M4019V, G-irradiated, Sigma Aldrich, USA) intravenously via the auricular vein using a glass syringe (2 mL, Socorex, Switzerland) with a butterfly needle (23G X ¾” 0.6 × 19 mm, 18 cm; Terumo, Belgium). Syringes and needles were flushed with 0.5–1.0 mL sterile physiological saline solution (0.9 g/L, Braun, Germany) before and after application. Clinical examinations, i.e. heart rate, rectal temperature and outer auricular skin temperature, were recorded by veterinary personnel prior to blood sampling before (0 h) and 3, 5, 8, 12, 24 and 48 h after the LPS injection, without animal immobilization and as soon as possible after entering the pen. The heart frequency (in beats per minute, BMP) was monitored with a stethoscope for large animals (Götze 65 mm, Hauptner und Herberholz, Germany), and the core and skin temperatures were measured with a digital rectal thermometer (GÉNIA VT-801RW5T, France) and an infrared camera (FLIR E6, Precision Technic Nordic, Norway), respectively. After the 48-h monitoring period, these LPS-exposed pigs continued in the dietary study until slaughtering at 80 days into the trial.

### Sampling

#### Blood

Blood samples were collected from all pigs (i.e. *N* = 16 per diet) at the beginning of the feeding trial (day 0) and after 16 and 77 days, and from all pigs in the 9-pig pens at 49–56 days into the trial (0 h) and 3, 5, 8, 12, 24 and 48 h after the LPS injection. Pigs were immobilized using a rope on upper jaw and blood samples were collected via the *vena jugularis externa* using 6-mL Vacuette Lithium Heparin tubes (Greiner Bio-One, Austria) with Venoject needles (20G × 1½’’UTW, USA). Lithium heparin blood was subjected to haematological analysis, whereas heparinized plasma was isolated by centrifugation at 3500 *g* for 15 min (Megafuge 1.0 R, Heraeus SEPATECH, USA) for subsequent clinical biochemistry, Se and VitE analyses. Samples were stored at – 20 °C or – 70 °C until the day of analysis.

#### Tissues

Tissue samples of skeletal muscles (*Musculus longissimus dorsi*, *Musculus semimembranosus* and diaphragm), myocardium (left ventricle), liver, kidney (cortex) and brain (frontal cortex) were collected from LPS-exposed pigs (*N* = 7–8 per diet) *post-mortem* after slaughtering at day 80 into the trial. For histopathological examination, subsamples of the *M*. *longissimus dorsi* and myocardium (ca. 15 × 10 × 5 mm; *N* = 7 per diet) were fixed in 4% neutral-buffered formaldehyde, cut into 5 µm thin sections and stained with haematoxylin and eosin. Tissue subsamples for Se analysis were collected in plastic vials and stored at – 20 °C, and for VitE analysis they were vacuum-packed and stored at – 70 °C.

### Analytical Methodology

#### Haematology and Clinical Biochemistry

Multi-parametric haematological (*N* = 7–9 per diet) and clinical biochemistry (*N* = 5–8 per diet) analyses are described in detail in section S2 of the supplementary information (SI).

#### Vitamin E Analysis

Vitamin E (as alpha-tocopherol) concentration in feed (*n* = 2 per diet), plasma (*N* = 7 per diet) sampled at 0, 16 and 77 days, and *post-mortem* tissues (myocardium, liver, *M*. *longissimus dorsi* and *M*. *semimembranosus*; *N* = 5 per diet) was determined by RP-HPLC [41]. Sliced frozen tissue and feed samples (1 g) were first homogenized with 1.15% KCl solution (9 mL) at 1000 rpm for 45 s (Potter–Elvehjem homogenizer), whereas plasma subsamples (100 µL) were vortex-mixed with 0.1 M phosphate buffer solution (pH 7.2, 100 µL) and 0.1% BHT in ethanol (10 µL). Homogenized samples (2 mL; 210 µL) were then vortex-mixed with ethanol (2 mL; 300 µL) and an internal standard aliquot (100 µL of 50 µg/mL delta-tocopherol), followed by hexane extraction (4 mL, 5 min; 400 µL, 1 min). After separation by centrifugation (4 min, 4500 rpm, Labofuge 400R; 3 min, 12,000 rpm, minicentrifuge Gusto), the hexane phase was transferred to new Eppendorf tubes and evaporated (50 °C) under nitrogen atmosphere. The residue was dissolved in methanol (0.5 mL; 0.25 mL) and 50 µL were used for analysis. Vitamin E was separated and detected at 40 °C using a HPLC Knauer system equipped with Chrompack Inertsil ODS-3, 5 µm column (250 × 4.6 mm) and precolumn (10 × 3.0 mm), 98% methanol mobile phase at 1.5 mL/min flow and UV and fluorescence detectors set to λ = 295 or 330 nm.

#### Total Selenium Analysis

Total Se concentration in feed (*n* = 3 per diet), tissues (*N* = 7–8 per diet) and plasma (*N* = 5–9 per diet) was determined by ICP-MS (Agilent 8900, Japan) following a similar methodology as in Falk et al. [31, 32, 36]. Before microwave acid digestion (ultrapure, sub-boiled HNO_3_, 260 °C, 25 min; UltraClave or UltraWave systems, Milestone), feed subsamples (0.17 ± 0.02 g; *n* = 24) were ground and homogenized, and tissues were freeze-dried for > 20 h and subsamples sliced (0.15 ± 0.07 g; *n* = 378). Plasma subsamples (0.51 ± 0.13 g; *n* = 492) were diluted with a mixture of butanol, EDTA, NH_3_ and Triton X-100 (1:10 V/V). Enriched ^74^Se (> 99.9%) was used as internal standard, and calibration standards were prepared from a Se ICP reference solution (1000 mg/L; Inorganic Ventures). Methods blanks (*n* = 5) and certified reference materials (1567a wheat flour and 1570a spinach, NIST, USA; Seronorm™ L-1 and L-2 serums, SERO, Norway) were prepared and analysed in the same manner as the samples for each analysis run. The accuracy of the overall method was 97 ± 4% (*n* = 15) and the reproducibility, evaluated by the relative standard deviation (RSD) of repeated measurements, was < 1% within an analysis run and < 3% between analysis days. The limit of quantification (LOQ = 10 times the standard deviation of the method blanks measured concentrations) was 7–21 µg Se/kg for feed and tissues, and 0.037–0.077 µg Se/L for plasma.

#### Selenium Speciation Analysis

The distribution of selenospecies in plasma (*N* = 5–9 per diet) was determined by HPLC (Agilent HP1260 liquid chromatograph, Agilent Technologies Inc., USA) in tandem with ICP-MS in time-resolved analysis mode (Agilent 8800, Japan; the same operating parameters as applied in Falk et al. [32]). Subsamples (0.40 ± 0.05 mL; *n* = 429) of thawed and homogenized plasma were first ultrafiltered (60 min, 14,000 *g*, 4 °C; 3 kDa Amicon 0.5 mL centrifugal filters, Merck, USA) to separate free Se and seleno amino acids (< 3 kDa, filtrate) from larger molecules such as selenoproteins (> 3 kDa, retentate). Inorganic Se (i.e. low molecular mass selenospecies such as selenite and H_2_Se; retention time (t_R_) ~ 0.7 min) and seleno amino acids (SeCys, t_R_ ~ 1.8–2.0 min; SeMet, peaks at t_R_ ~ 1.5–1.7 and 7.5–9.5 min) were separated using an Atlantis T3 column (4.6 × 75 mm, 3 µm; Waters) and a 7.5% (V/V) methanol mobile phase containing 0.1% (V/V) of HFBA in isocratic mode (1.5 mL/min flow, 25 μL sample loop). Proteins, i.e. the selenoproteins GPx3 and SelP and the Se-containing protein SeAlb, were separated using HiTrap Heparin and HiTrap Blue HP columns (1 mL; GE Healthcare, Uppsala, Sweden) connected in-line via a 10-port valve and 0.05 and 1.5 M ammonium acetate solutions as retention and elution mobile phases, respectively (1.0 mL/min flow, 25 µL sample loop). GPx3 was not retained in the columns and eluted 1.0–1.8 min after sample injection. At 3 min, the mobile phase was changed, and the flow passed only through the HiTrap Heparin column to elute the retained SelP (t_R_ ~ 5.1 min). SeAlb was sequentially eluted at ~ 9.1 min by switching the flow to pass again through both columns after 8 min. Representative chromatograms obtained with these methodologies are shown in Fig. S4.

Integrated chromatogram peak areas were converted to Se concentration (in µg/L) using seleno-l-methionine (≥ 98% (TLC), Sigma-Aldrich) and seleno-l-cysteine (95%, Sigma-Aldrich) standard solutions measured under the same elution conditions as for the selenospecies. Blank samples (mobile phase solutions) and standard solution checks were run every 10 samples to assess signal drift. The limit of quantification (as the Se concentration necessary to yield a net signal equal to 10 times the standard deviation of the background signal) was 0.024 µg Se/L for inorganic Se, SeCys and SeMet, and 0.07, 1.22 and 2.67 µg Se/L for GPx3, SelP and SeAlb, respectively. Additionally, two human serum certified reference materials (BCR 637 and BCR 639; EC-JRC-IRMM, Belgium) were prepared and analysed in triplicate in the same manner as the samples to assess reproducibility and accuracy. The overall method from sample preparation to analyses presented the necessary analytical figures-of-merit for the determination of selenospecies in plasma, i.e. < 2.5% RSD for instrumental repeatability, ~ 4% and ~ 10% RSD for method reproducibility within an analysis run and between analysis days, respectively, and 87–120% recovery of Se (as the sum of selenospecies relative to the certified total Se concentration).

#### Histopathology

Sections from the *M*. *longissimus dorsi* and left ventricle myocardium (*N* = 7 per diet) of LPS-exposed pigs were examined blindly by light microscopy. Histopathological lesions were graded on a semi-quantitative scale of severity from 0 to 4, including half-step grading, based on degeneration, regeneration and necrosis of muscle fibres and infiltration of inflammatory cells (counted as lesions per 100 magnification view). For myocardial samples, vacuolation of myocytes was also taken into account. Grading scales were set for the *M*. *longissimus dorsi* and myocardium, respectively, as follows: 0 for none or minor changes with ≤ 2 and ≤ 5 lesions; 1 for mild changes with 2–5 and 5–10 lesions; 2 for moderate changes with 5–10 and 10–20 lesions; 3 for major changes with 10–20 and 20–30 lesions; and 4 for severe changes with ≥ 20 and ≥ 30 lesions.

### Statistical Analyses

Prior the start of the trial, the sample size was determined by power analysis using the SAS 9.4 software. This resulted in 128 animals (*N* = 16 replicates/treatment) in a completely randomized block design so to meet the generally accepted minimum statistical power of 1-β = 0.8. This group size should allow to detect differences in total Se, selenospecies and VitE levels in plasma and tissue samples. However, in order to find significant dietary effects in weight gain or feed efficiency, higher than 32 pigs/treatment would have been necessary (calculation based on average differences and standard deviations for daily BW gain (50 and 120 g/day) and feed efficiency (0.03 and 0.15 FEn/kg) reported for previous feeding trials at the same facility). Since this study focused on identifying differences in essential nutrient levels, a trade-off in favour of molecular measurements was made and the group size was set to 16 animals per diet.

Statistical analyses were performed on production parameters, clinical examinations, haematological and biochemical, VitE, total Se and selenospecies data using Minitab 17 statistical software (diet groups) or Microsoft Office Excel (time points; correlations) and on histopathology scores using PyCharm 2020.2.1 (JetBrains) with the scipy.stats module (SciPy v1.6.0). For intergroup evaluation, data sets were tested for normal distribution (Ryan-Joiner test) and homogeneity of variance (equal variance, Levene’s test) and outliers were identified (Grubb’s test). A one-way ANOVA test (Tukey pairwise comparison) was then performed to determine whether variable averages were significantly different between diet groups within a given time point or tissue type. The significance level was initially set to α = 0.05, but since 8 diet groups were tested against each other for each variable and an eventual time effect could occur in the LPS challenge study, a Bonferroni correction was applied. The resulting Bonferroni critical value of *p* < 0.0018 was thus taken as significant when comparing diet treatments. Additionally, to evaluate differences due to potential pen size effects, comparisons between pens were also performed in averages of production parameters (all pens vs. all) and of clinical examinations, blood parameters, VitE, total Se and selenospecies (between the two pens within a diet group).

For time variations, relative change in averaged variables for each diet group (VitE, total Se and selenospecies) or for all diet groups together (haematology and biochemistry) at any given time point with respect to initial conditions was evaluated for significant differences by the paired *t* test (two-tailed distribution; critical values were *p* < 0.05 for significant trends and Bonferroni corrected *p* < 0.0018 for significant changes).

The strength of correlation between the VitE concentrations in feed and the measured VitE and Se levels in plasma and tissues, and between the latter, was evaluated based on Pearson correlation coefficients (*r*) as follows: *r* > 0.8 indicates a very strong positive correlation, *r* = 0.6–0.8 indicates a strong positive correlation, *r* = 0.4–0.6 indicates a moderate positive correlation, *r* = 0.2–0.4 indicates a weak positive correlation and *r* values closer to 0 indicates little to no positive correlation. Differences in lesion scores for each diet group were statistically evaluated using the Mann–Whitney rank test function and a Bonferroni critical value of *p* < 0.0018.

## Results

### Dietary *S*tudy

#### Production Parameters

The BW increased linearly over time for all pigs independently of diet treatment or pen size (Figs. S1–S2; weekly averages in Table S2). By the end of the 80-day trial, the pigs had gained in average ~ 89 kg at an average linear rate of 1.14 kg/day (R^2^ = 0.930) with no significant differences in BW averages between the diet groups neither between the different pens (Table S1). The feed intake and number of visits to the feeder per animal per day presented a natural variability between individuals but without clear differences between pens or diet groups (weekly averages in Tables S3–S4).

#### Haematology and Clinical Biochemistry

The levels of red and white blood cells and related parameters, enzymes, proteins and trace elements in all the pigs were within reference levels at the initiation of the trial (day 0) and changes during animal growth were in general not significantly different between diet groups. A detailed description of change over time, average levels for all diet groups and significance test results can be found in section S2 and Table S5.

#### Total Selenium and Vitamin E in Plasma

**Fig. 1:**
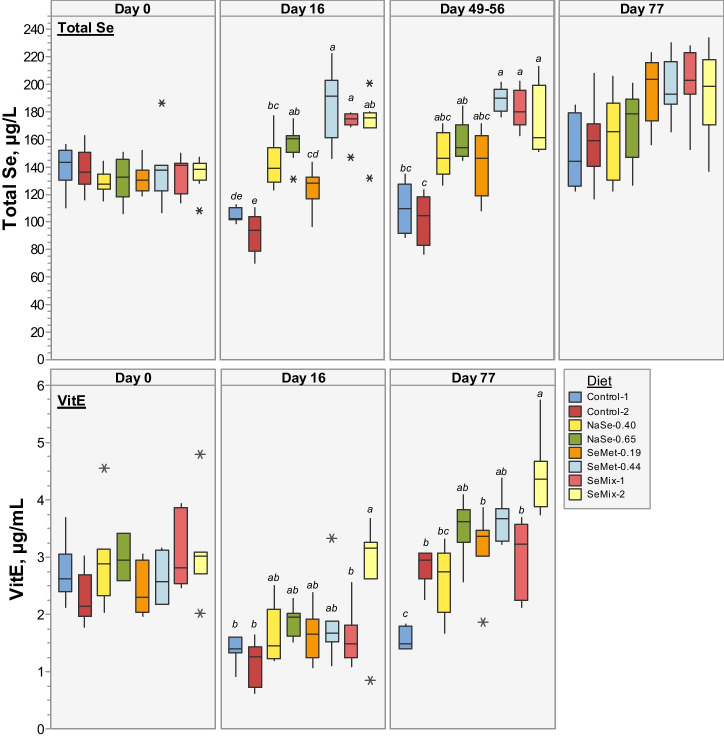
Boxplots of total Se (*top*) and vitamin E (*bottom*) concentrations in plasma of pigs fed non-Se-supplemented (Control) and NaSe and/or SeMet enriched diets (Table 1) at the start of the trial (day 0) and 16, 49–56 and 77 days into the trail (*N* = 5–9 per diet). Box data within a given time point that do not share a grouping letter have significantly different averages (one-way ANOVA, Tukey pairwise, 99.82% confidence level). Average concentrations, associated standard deviations, and significance test grouping are presented in Table S6 in the SI

The initial total Se and VitE concentrations in plasma were not significantly different between all diet groups (day 0 in Fig. 1, *p* > 0.8 and > 0.2, respectively; Table S6). For the Control-1 and Control-2 diet groups, i.e. non-Se-supplemented diets, the total Se plasma concentration decreased from ~ 140 to ~ 100 µg Se/L at day 16, remained constant for another 33–40 days and increased to initial levels by the end of the trial (*p* = 0.16, 0.10; Fig. 1). In pigs fed NaSe-0.40 and SeMet-0.19, the total Se in plasma was similar to the initial concentrations at day 16, followed by a slight increase of 8–16% at days 49–56. This increasing trend continued until day 77 (24–49% respect to day 0), becoming significant for SeMet-0.19 (*p* = 0.000076; *p* = 0.025 for NaSe-0.40; Fig. 1). Increasing the Se-supplementation (NaSe-0.65, SeMet-0.44, SeMix-1, SeMix-2) resulted in 20–37% significantly higher plasma Se concentrations already at day 16 (all *p* < 0.00035), followed by a slow increase of an additional 9–20% until day 77 (all *p* < 0.0011 respect to day 0; Fig. 1). Nevertheless, SeMet-0.44, SeMix-1 and SeMix-2 diets resulted in 9–30% higher total Se concentrations in plasma than NaSe-0.40 or NaSe-0.65 diets at any time point of the trial.

The VitE plasma levels decreased by 31–49% (all *p* < 0.0015) during the first 16 days in pigs fed the diet with ~ 25 mg VitE/kg (i.e. Control-1) or the diets with ~ 50 mg VitE/kg (i.e. NaSe-0.40, NaSe-0.65, SeMet-0.19, SeMet-0.44, SeMix-1), but only by 8% (*p* = 0.36) in pigs fed the SeMix-2 diet containing ~ 100 mg VitE/kg (Fig. 1). Yet, neither diet group fell below 1.0 or 1.5 µg/mL, which has been considered as distinctly deficient in Norway and Denmark, respectively [2, 42]. By the end of the trial (day 77), the VitE levels of pigs fed Control-1 diet had not recovered (*p* = 0.22 respect to day 16), whereas those from pigs fed Control-2, NaSe-0.40 and SeMix-1 diets reached initial plasma concentrations (*p* = 0.71, 0.24, 0.83 respect to day 0). In comparison, the NaSe-0.65, SeMet-0.19 and SeMet-0.44 diets resulted in 16–37% higher VitE plasma levels (*p* = 0.058, 0.029, 0.0048), and up to a 45% increase was observed with SeMix-2 diet (*p* = 0.015). The VitE concentration in the feed was strongly postively correlated to the VitE plasma levels by the end of the trial (*r* = 0.17, 0.59 and 0.73 for day 0, 16 and 77, respectively), but only weak to moderate positively correlated to the Se plasma levels (*r* =  − 0.06, 0.45 and 0.29 for day 0, 16 and 77, respectively). Between diets with the same VitE and Se concentrations in the feed (i.e. ~ 50 mg VitE/kg and ~ 0.4 mg Se/kg), SeMet-0.44 resulted in 42% and 23% higher VitE plasma levels by day 77 than NaSe-0.40 and SeMix-1 diets, respectively (*p* = 0.007, 0.024); the latter two showing similar VitE levels that were not significantly different (*p* = 0.98, 0.99) to the non-Se-supplemented diet Control-2 (Fig. 1, Table S6). Plasma of pigs fed the same VitE concentration (~ 50 mg/kg) but ~ 0.2 mg/kg higher Se concentration (i.e. NaSe-0.65 *vs. *NaSe-0.40 and SeMet-0.44 *vs.* SeMet-0.19) showed higher average VitE plasma concentrations by the end of the trial (36% difference between NaSe-enriched diets, *p* = 0.031; 14% for SeMet-enriched diets, *p* = 0.74; Fig. 1, Table S6). It is also worth noting that the low SeMet-0.19 diet resulted in VitE plasma concentrations that were 24% higher than with the low NaSe diet and similar to those of pigs fed the high NaSe diet (*p* = 0.32, 0.95; Fig. 1, Table S6). Overall, there was a weak to moderate positive correlation between the concentrations of VitE and Se in plasma (*r* = 0.15, 0.40 and 0.32 for day 0, 16 and 77, respectively).

#### Distribution of Selenospecies in Plasma

**Fig. 2:**
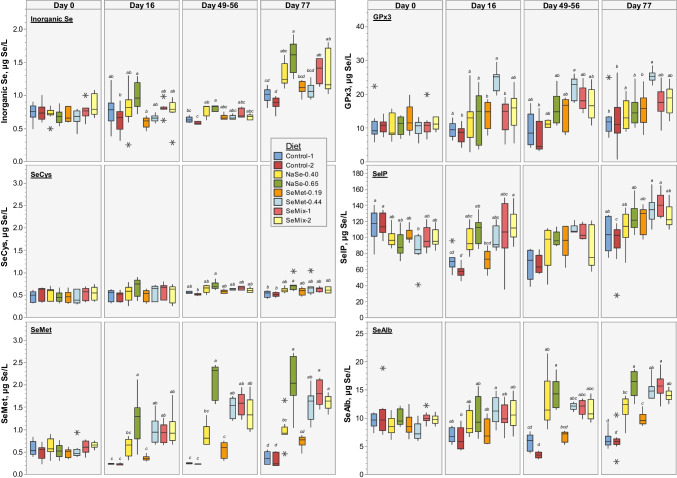
Boxplots of Se concentration (in µg/L) associated to selenospecies (*left*—inorganic Se, SeCys, SeMet; *right*—GPx3, SelP, SeAlb) in plasma of pigs (*N* = 5–9 per diet) fed non-Se-supplemented (Control) and NaSe and/or SeMet enriched diets (Table 1) at the start of the trial (day 0) and at day 16, 49–56 and 77 into the trial. Box data within a given time point that do not share a letter have significantly different averages (one-way ANOVA, Tukey pairwise, 99.82% confidence level). Average concentrations, associated standard deviations and significance test grouping are presented in Table S7 in the SI

Determination of Se associated to selenospecies in plasma showed low concentrations of inorganic Se and seleno amino acids for all pigs (< 2.5 µg Se/L), yet significant differences between diet groups were observed by the end of the trial (Fig. 2; Table S7). Over the first 16 days of the trial, the inorganic Se and SeCys levels were relatively constant and presented high variability between pigs within each diet group, followed by a slight increase at days 49–56 and 77 (0.05 > *p* > 0.00001), especially for inorganic Se, with the highest levels determined in plasma of pigs fed diets containing selenite, i.e. NaSe-0.65 > NaSe-0.40 ~ SeMix-1 ~ SeMix-2 ~ SeMet-0.44 > SeMet-0.19 > Control-1 ~ Control-2. On the other hand, the SeMet levels in the first 16 days decreased by 55% in pigs fed the Control diets (*p* = 0.0016, 0.0005), by 20% in pigs fed SeMet-0.19 diet (*p* = 0.0065) and remained relatively constant for the NaSe-0.40 diet group (*p* = 0.44). During the remaining trial period, no clear changes were observed in the Control groups (*p* = 0.035, 0.12), whereas the SeMet levels increased slowly with both SeMet-0.19 and NaSe-0.40 diets (*p* = 0.043, 0.019 respect to day 0; no significant difference between these two diets; Fig. 2, Table S7). Increasing the SeMet concentration in the feed (SeMet-0.44, SeMix-1, SeMix-2) increased the SeMet levels in plasma by 60–100% already at 16 days (*p* = 0.0002, 0.0058, 0.0097), reaching up to 150–220% higher concentrations by the end of the trial (*p* < 0.00004; no significant differences between these three diets; Fig. 2, Table S7). The NaSe-0.65 diet resulted in an increase of 250–270% respect to day 0 after 49–56 and 77 days (*p* = 0.00008), but the SeMet levels were not significantly different to those determined in SeMet-0.44, SeMix-1 and SeMix-2 diets groups (Fig. 2, Table S7).

At the beginning of the trial, SelP was the most abundant selenospecies in plasma of all pigs (~ 100 µg Se/L), whereas GPx3 and SeAlb levels were 1 order-of-magnitude lower (Fig. 2). In the Control diet groups, no clear changes in the GPx3 levels were observed during the trial period (all *p* > 0.5), while SelP and SeAlb levels decreased by half after 16 days (*p* < 0.004), with no further changes (SeAlb; *p* < 0.013) or a recovery to initial levels (SelP; *p* > 0.06) by the end of the trial. Sodium selenite-supplemented diets (NaSe-0.40, NaSe-0.65) resulted in GPx3 levels close to the Control diets but lower than those determined in plasma of pigs fed SeMet-0.19 and both SeMix diets, which increased linearly over time (up to 75% increase by day 77, *p* < 0.03 respect to day 0). Notably, the GPx3 levels in plasma of pigs fed SeMet-0.44 diet increased by ~ 150% already after 16 days (*p* = 0.00002) and remained constant during the trial period (*p* = 0.000001), resulting in the highest levels among all diet groups (significantly different to the Control, NaSe and SeMet-0.19 diets; Fig. 2, Table S7). The SelP and SeAlb levels in pigs fed either NaSe, SeMet-0.44 or either SeMix diet also increased overtime, with 15–50% (0.017 > *p* > 0.00004) and 35–95% (0.0036 > *p* > 0.000003) higher concentrations by day 77, respectively. Although no significant differences were observed between these diet groups, the average levels differed significantly compared to control diets (Fig. 2, Table S7). Low SeMet levels in the feed (SeMet-0.19) resulted in a decrease of SelP and SeAlb during the first 16 days (*p* = 0.00027, 0.011) followed by a slow recovery to above initial levels by the end of the trial (*p* = 0.022, 0.22). At day 77, the average SelP and SeAlb concentrations were similar to plasma levels of pigs fed NaSe-0.40 diet and notably higher than those resulting from the Control diets (significant differences for SeAlb; Fig. 2, Table S7).

#### Total Selenium and Vitamin E in Tissues

**Fig. 3:**
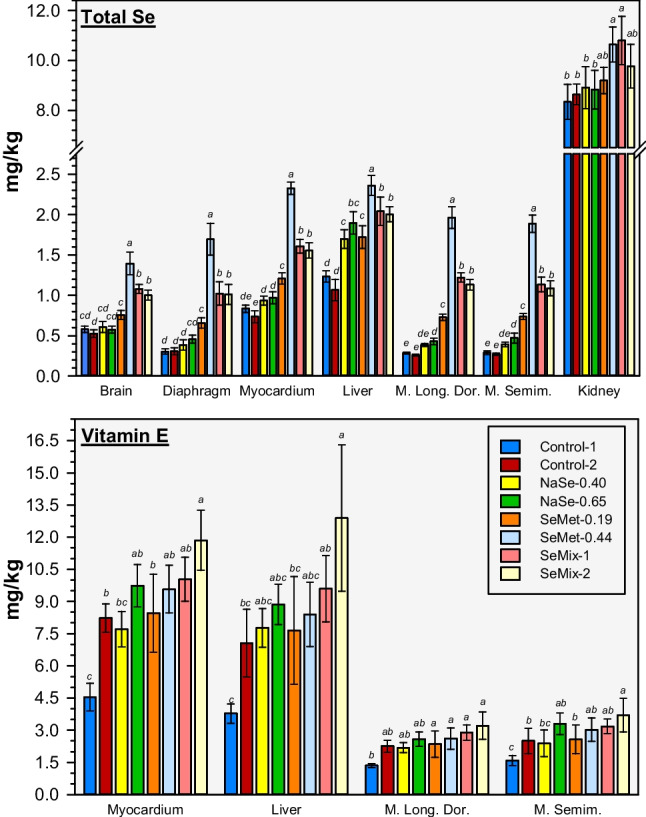
Bar charts of average total Se (*top*) and vitamin E (*bottom*) concentrations in *post-mortem* tissues (in mg/kg dry matter) from pigs fed non-Se-supplemented (Control) and NaSe and/or SeMet-enriched diets (Table 1) at the end of the 80-day dietary study (*N* = 5–8 per diet; error bars represent 1σ). Muscles name abbreviations refer to *musculus longissimus dorsi* (M. Long. Dor.) and *musculus semimembranosus* (M. Semim.). Bar data within a given tissue that do not share a letter have significantly different average concentrations (one-way ANOVA, Tukey pairwise, 99.82% confidence level). Average concentrations, associated standard deviations, and significance test grouping are presented in Table S9 in the SI

The highest total Se concentration in tissues collected *post-mortem* was determined in kidney (8–11 mg/kg), while the liver, skeletal muscles (diaphragm, *M*. *longissimus dorsi*, *M*. *semimembranosus*), myocardium and brain contained on average 5 to 30 times lower Se concentrations (Fig. 3). Sodium selenite-supplemented diets (NaSe-0.40, NaSe-0.65) resulted in similar concentrations to the Control diets in all tissues, except for the liver that presented significantly higher average Se concentrations (40–80% higher; Fig. 3, Table S9). Kidney and liver of pigs fed SeMet-0.19 diet showed similar Se concentration to the Control and NaSe diet groups, but the skeletal muscles (45–180%) and the myocardium and brain (25–65%) presented significantly higher Se concentrations (Fig. 3, Table S9). Increasing the dietary SeMet concentration (SeMet-0.44) resulted in significantly higher Se concentrations in all tissues than those resulting from the Control and NaSe diets (15–30% higher in kidney, 25–120% higher in liver, 130–220% in myocardium and brain, 155–650% in muscles; Fig. 3, Table S9). The mixed source feed (SeMix-1, SeMix-2) resulted in liver, skeletal muscles, heart and brain Se concentrations that were significantly lower (15–40%) than those measured in tissues of pigs fed the SeMet-0.44 diet but significantly higher (10–90% in liver, 60–120% in heart and brain, 120–370% in muscles) than those of pigs fed the Control or NaSe diets (Fig. 3, Table S9). Average Se concentrations in kidney of both SeMix diet groups were comparable to those from SeMet diets but 10–30% higher than those from the Control and NaSe diets (only differing significantly for SeMix-1; Fig. 3, Table S9).

For all diet groups, VitE concentrations in the liver and myocardium were 2.5–4 times higher than in the skeletal muscles (Fig. 3). The VitE concentrations in tissues from pigs fed Control-1 diet (~ 25 mg VitE/kg; no-Se-supplemented) were 35–70% lower than for any other diet group (significant differences observed for SeMix-1, SeMix-2, SeMet-0.44 and NaSe-0.65 diets; Fig. 3, Table S9). Doubling the VitE concentration in feed with no Se supplementation (i.e. Control-2 *vs*. Control-1) resulted in 60–90% higher VitE levels in all tissues (significantly different for myocardium; Fig. 3, Table S9); whereas in pigs fed Se-supplemented diets (i.e. SeMix-2 *vs.* SeMix-1), the average VitE concentrations were 10–15%, 20% and 35% higher in muscles, myocardium and liver, respectively, with higher VitE in feed (no significant differences between these diet groups; Fig. 3, Table S9). Overall, the VitE concentration added to the feed was moderately to strongly correlated to the VitE accumulated in any of the tissues (*r* = 0.40, 0.74, 0.55 and 0.44 for liver, myocardium, *M*. *longissimus dorsi* and *M*. *semimembranosus*, respectively) and very strongly correlated to the Se accumulated in liver, *M. longissimus dorsi* and *M. semimembranosus* tissues (*r* = 0.83, 0.82 and 0.99, respectively; *r* = 0.33 for myocardium). Increasing the Se concentration in the diets with ~ 50 mg VitE/kg (i.e. NaSe-0.65 *vs.* NaSe-0.40 and SeMet-0.44 *vs. *SeMet-0.19) resulted in 10–40% higher average VitE concentrations, with 0.3–0.9 mg/kg more in liver and skeletal muscles and up to 2 mg/kg more in the myocardium (no significant differences between these diet group pairs; Fig. 3, Table S9). Moreover, between the three diets containing ~ 0.40 mg Se/kg and ~ 50 mg VitE/kg, the SeMet-0.44 diet resulted in 10–30% higher VitE concentrations in skeletal muscles, myocardium and liver than those from pigs fed the NaSe-0.40 diet, but slightly lower (5–10%) than those from pigs fed the SeMix-1 diet (not significant diet group differences for any of the tissues; Fig. 3, Table S9). Overall, there was a moderate correlation between the tissue concentrations of Se and VitE (*r* = 0.50, 0.57, 0.47 and 0.39 for myocardium, liver, *M*. *longissimus dorsi* and *M*. *semimembranosus*, respectively) and, interestingly, the SeMet-0.44 diet resulted in a stronger (*r* = 0.65–0.71) positive Se-VitE correlation in myocardium and skeletal muscles than any other diet.

### LPS Challenge Study

#### Clinical Examinations

The intravenous LPS application resulted in an increase in heart rate and rectal temperature and a decrease of auricular skin temperature in all pigs (no significant differences between diet groups; Fig. S3). The average heart rate (*N* = 69) quickly raised from the initial 121 ± 19 BPM to 130 ± 21 and 141 ± 16 BPM after 3 and 5 h, respectively, returning close to initial values at 8 to 12 h after the LPS injection (129 ± 12 and 125 ± 16 BPM, respectively). The initial and final heart rates were generally higher than the expected values for pigs at this age (i.e. 78–85 BPM), which may indicate an increased stress level due to handling. Nonetheless, the average core temperature followed a similar time trend, increasing from 39.3 ± 0.3 °C before injection to 41.2 ± 0.4 °C after 3 h, continued at 40.7 ± 0.5 °C at 5 h, and decreased to normal core temperatures after 8 h (39.4 ± 0.3 °C) without further changes (39.3 ± 0.3 °C at 12 h). Contrastingly, the average outer auricular skin temperature was up to 8° lower after 3 and 5 h since the LPS injection (32.3 ± 3.2 °C *vs.* 24.0 ± 4.1 °C and 26.7 ± 5.6 °C, respectively) and did not reach initial values after 8 nor 12 h (29.9 ± 5.1 °C and 30.1 ± 4.8 °C, respectively).

#### Haematology and Clinical Biochemistry

Plasma parameters after the LPS injection showed no significant differences between diet groups, except for the LDH levels of Control-1 diet group that increased between 5 and 12 h up to levels 30% to 75% higher than in plasma of pigs from the other diet groups (significantly different to NaSe-0.40, SeMet-0.44 and SeMix-1; similar values to SeMe-0.19). Among all the measured blood parameters, it is worth mentioning the significant decrease of WBC and related parameters within 8 h after the LPS injection as well as the RBC, Hgb, Hct and Fe that reached minimum values between 8 and 24 h. Average levels and changes over time are described in detail in section S2 and Table S5.

#### Total Selenium and Selenospecies Distribution in Plasma

The average total Se concentration tended to decrease for all diet groups in the first 8–12 h after receiving the LPS injection, but differences with respect to initial values were only significant in plasma of pigs fed NaSe-0.65 and SeMix diets (*p* = 0.0017, 0.042, 0.0012; Fig. S5). After 24–48 h, the total Se concentrations in plasma from these pigs, as well as from those fed NaSe-0.40 and SeMet-0.44 diets, increased significantly above the levels prior to the injection (0.050 > *p* > 0.0012; Fig. S5). During the 48-h monitoring period, the differences between diet groups (Table S8) were the same as those observed for total Se plasma concentration during the dietary study at days 49–56 (Table S6).

**Fig. 4:**
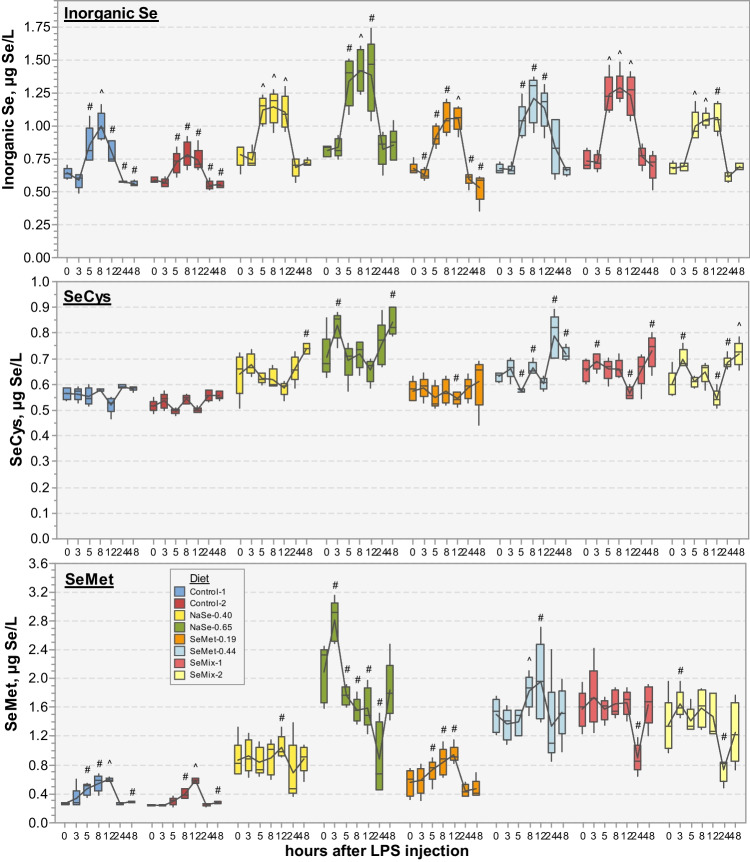
Boxplots of Se concentration (in µg/L) associated to inorganic Se, SeCys and SeMet in plasma of pigs (*N* = 5 per diet) fed with non-Se-supplemented (Control) and NaSe and/or SeMet enriched diets (Table 1) before and 3, 5, 8, 12, 24 and 48 h after receiving an injection of LPS (2 µg/kg BW) at 49–56 days into the trial (x-axes, non-linear scale). Box plots are connected by the average at each time point (solid line), whose significance difference respect to time 0 h is indicated with # for *p* < 0.05 and ^ for *p* < 0.0018 (paired *t* test, two-tailed distribution). Average concentrations, associated standard deviations, and significance test grouping between diet groups within a given time point are presented in Table S8 in the SI. The corresponding total Se concentrations over the same time period can be found in Figure S5 and Table S8 in the SI

Inorganic Se levels increased significantly by 25–75% for all diet groups 5–12 h after the LPS injection (0.025 > *p* > 0.00005), only returning to initial values at 24 h (0.87 > *p* > 0.039; Fig. 4). No changes over time were observed in the SeCys levels of pigs fed control or SeMet-0.19 diets (0.94 > *p* > 0.057), but fluctuations were observed in plasma of pigs fed the other Se-supplemented diets, with a significant increase of 10–25% by 24–48 h (0.037 > *p* > 0.00011; Fig. 4). Contrastingly, the SeMet levels in plasma of Control-1, Control-2 and SeMet-0.19 fed pigs gradually increased between 3 and 12 h after the LPS injection to values 70–145% higher than the initial levels (0.039 > *p* > 0.00002). A small but significant increase of 20–35% was also observed for NaSe-0.65 and SeMet-0.44 fed pigs occurring, respectively, more rapidly between 0 and 3 h (*p* = 0.015) and 5 and 8 h (*p* = 0.00056). No significant changes were observed during the first 12 h for the NaSe-0.40 or for the first 24 h for the SeMix diet groups (0.65 > *p* > 0.33). In all diet groups, SeMet levels drastically dropped at 24 h, either to initial levels that remained constant up to 48 h (Control-1, Control-2, SeMet-0.19; *p* = 0.36, 0.30, 0.14) or to lower values (11–20% for NaSe-0.40, SeMet-0.44, *p* = 0.51, 0.61; 44–58% for NaSe-0.65, SeMix-1, SeMix-2, *p* = 0.010, 0.005, 0.032) that recovered by 48 h (0.91 > *p* > 0.22; Fig. 4, significant differences between groups in Table S8).

**Fig. 5:**
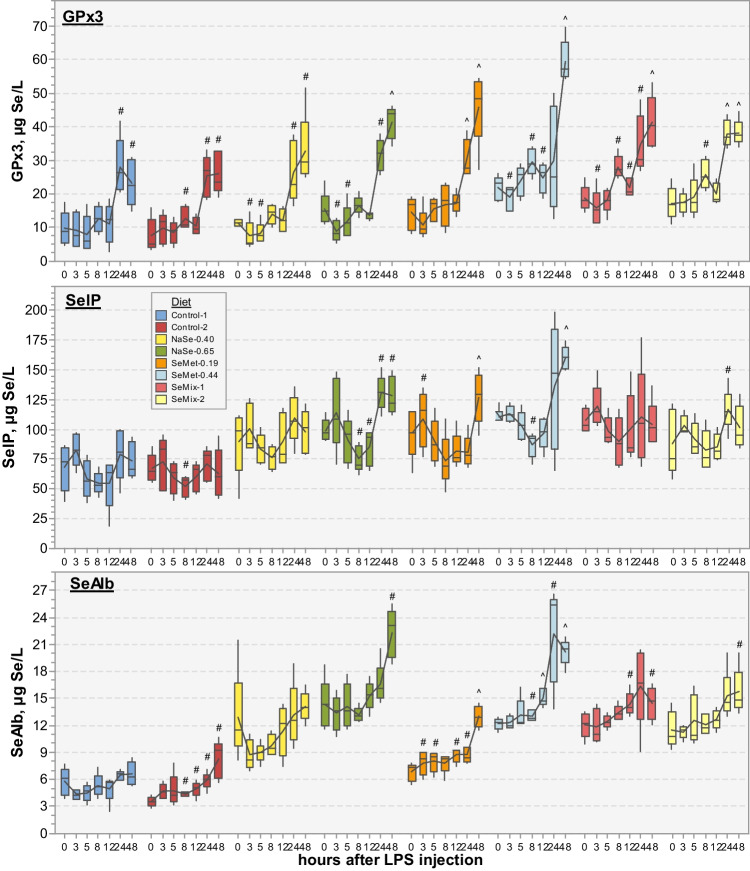
Boxplots of Se concentration (in µg/L) associated to GPx3, SelP and SeAlb in plasma of pigs (*N* = 5 per diet) fed with non-Se-supplemented (Control) and NaSe and/or SeMet enriched diets (Table 1) before and 3, 5, 8, 12, 24 and 48 h after receiving an injection of LPS (2 µg/kg BW) at 49–56 days into the trial (x-axes, non-linear scale). Box plots are connected by the average at each time point (solid line), whose significance difference respect to time 0 h is indicated with # for *p* < 0.05 and ^ for *p* < 0.0018 (paired *t* test, two-tailed distribution). Average concentrations, standard deviations, and significance test grouping between diet groups within a given time point are presented in Table S8 in the SI. The corresponding total Se concentrations over the same time period can be found in Figure S5 and Table S8 in the SI

For all diet groups, the average GPx3 levels in plasma (Fig. 5) either remained constant or decreased slightly during the first 5 h after the LPS injection (0.71 > *p* > 0.040), followed by a small increase after 8–12 h (significant for SeMet-0.44 and SeMix diet groups with 0.029 > *p* > 0.0066; 0.87 > *p* > 0.17 for the Control, NaSe and SeMet-0.19 diet groups), which by 24 h had significantly escalated to values 85–240% higher than initial levels (0.050 > *p* > 0.0010). These high GPx3 plasma levels stayed constant for control and SeMix-2 diet groups, but continued to increase in pigs fed either NaSe, SeMet or SeMix-1 diets. None of the diet groups returned to initial GPx3 plasma levels within the 48 h after the LPS injection (0.0031 > *p* > 0.00013). SelP plasma levels (Fig. 5) decreased by 5–25% for all diet groups between 3 and 8 h after the LPS injection (0.70 > *p* > 0.12), followed by a recovery between 12 and 24–48 h reaching (Control-1, Control-2, NaSe-0.40, SeMix-1; 0.98 > *p* > 0.16) or significantly surpassing by 15–45% (NaSe-0.65, SeMet-0.44, SeMix-2; 0.042 > *p* > 0.00032) the initial SelP levels, with exception of the SeMet-0.19 diet group levels that remained low up to 24 h after the LPS injection (*p* = 0.62 at 24 h; *p* = 0.0015 at 48 h). With regards SeAlb plasma levels (Fig. 5), no significant changes were observed for the first 5–12 h for control and NaSe-0.65 diet groups (0.86 > *p* > 0.074), whereas levels decreased by 30% within the first 3 h and remained low up to 8 h for the NaSe-0.40 fed pigs (*p* > 0.14) or gradually increased by 10–30% during the first 12 h for pigs fed SeMet and SeMix diets (0.0098 > *p* > 0.00017). Between 12 and 48 h, SeAlb plasma levels of all diet groups continued to increase, reaching 15–35% (Control-1, NaSe-0.40, SeMix-1, SeMix-2; *p* = 0.50, 0.55, 0.057, 0.042), 55% (NaSe-0.65; *p* = 0.028) or 65–90% (Control-2, SeMet-0.19, SeMet-0.44; *p* = 0.0057, 0.000047, 0.000070) higher values than those measured before the LPS injection. Significant differences between diet group averages can be found in Table S8.

#### Histopathological Evaluation

**Fig. 6:**
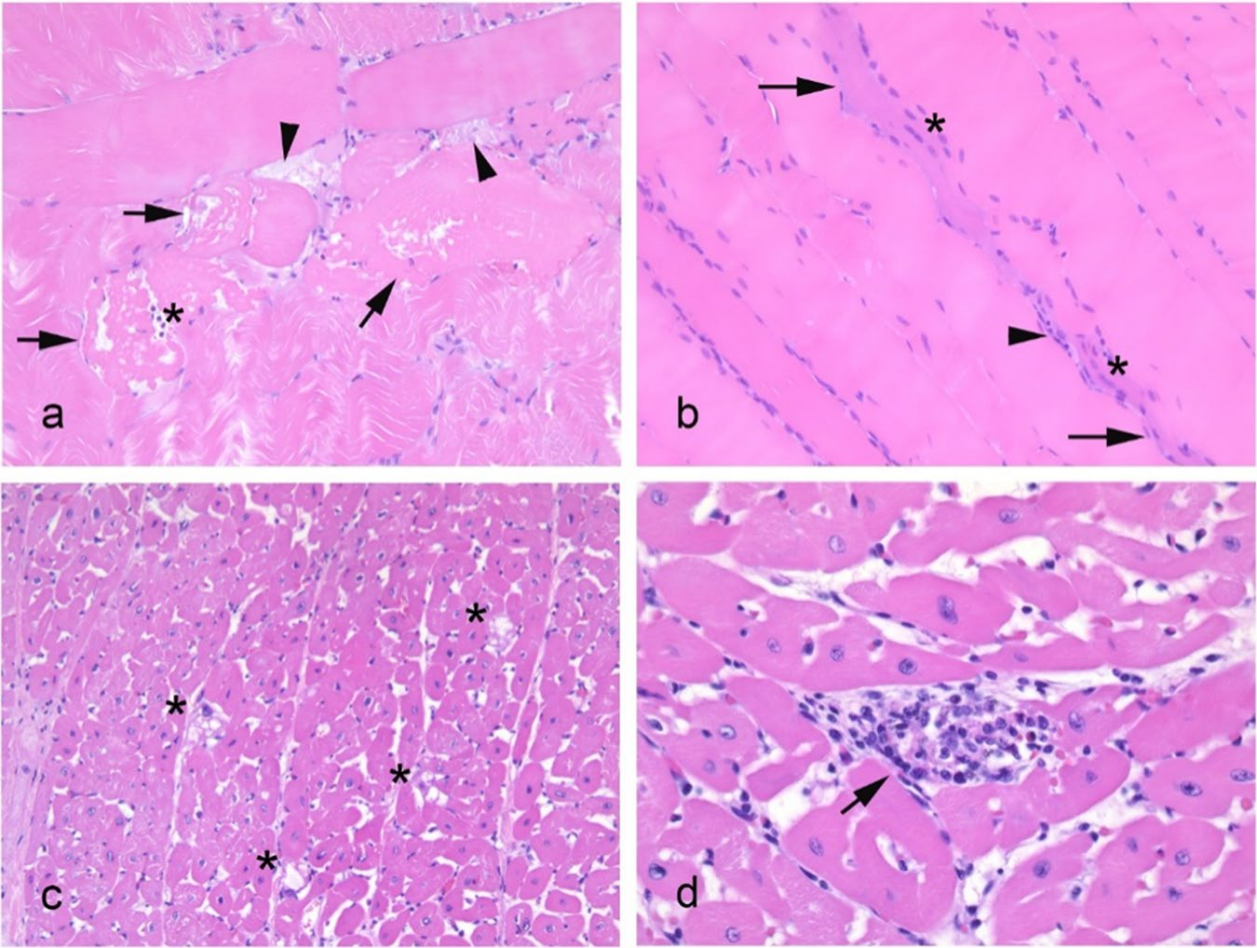
Representative histopathological changes in *post-mortem* tissues of LPS-exposed pigs: **a** *M. longissimus dorsi* with disintegrating fibres and loss of striation (arrows) and influx of inflammatory cells (asterisk) and extracellular proteinaceous material (arrowheads); **b** a regenerating, shrunken and basophilic striated muscle fibre (arrows) with centrally placed nuclei (asterisks) among normal *M. longissimus dorsi* fibres; **c** vacuolated myocytes (asterisks) in myocardium tissue; **d** a degenerated myocardial fibre (arrow) invaded by mononuclear inflammatory cells. Images correspond to tissues isolated from pigs fed the Control-1 (**b**), NaSe-0.40 (**a**, **d**) or NaSe-0.65 (**c**) diets

By the end of the trial, a spectre of small focal lesions was observed in muscle tissues of LPS-exposed pigs (Fig. 6). There was evidence of a multiphasic reaction in *M*. *longissimus dorsi* sections, with lesions including acute degenerative changes (as loss of striation and swelling and fragmentation of muscle fibres, sometimes with extracellular proteinaceous material; Fig. 6a) and reparative processes (including influx of inflammatory cells, reactive satellite cells, rowing of centrally placed myocyte nuclei and small basophilic fibres; Fig. 6b). For many pigs, the main lesion in the myocardium was vacuolar degeneration of myocytes with pleomorphic and vesicular nuclei (Fig. 6c). Subacute focal changes were also present, appearing in most cases with homogenous eosinophilic cytoplasm and infiltration of mononuclear cells (Fig. 6d). Nonetheless, the tissue evaluation showed a variation in lesion scores between individuals within a diet group (Fig. S6) and no statistical differences when comparing diet groups. Scores ranged from none or minor to mild changes in the *M*. *longissimus dorsi* from pigs fed SeMix-2 and SeMet-0.44 diets and in the myocardium of pigs fed either Se-enriched diet (i.e. NaSe, SeMet or SeMix). Pigs fed either control diet presented up to moderated changes in both tissues, as did the *M*. *longissimus dorsi* of pigs fed the NaSe, SeMet-0.19 and SeMix-1 diets. No pigs presented major or severe changes in any of the two muscle sections examined.

## Discussion

At the initiation of the trial (day 0), no significant differences in plasma levels of Se, including selenospecies, and VitE were observed between diet groups (Figs. 1 and 2, Tables S6 and S7), confirming comparable initial conditions among the pigs. As expected from the power calculations and the somewhat variable effects on pig growth performance reported in the literature [36, 43, 44], the production parameters were not significantly different between diet groups throughout the 80-day feeding period (Figs. S1–S2, Tables S1–S4). It is worth noting that the individual records of BW gain, feed intake and visits to the feeder allowed to confirm there was no fighting or hierarchy affecting the drinking and eating behaviour of single individuals within a given pen, enabling us to investigate changes in blood parameters and essential nutrient levels and speciation due to diet composition (the main focus of this study). Only marginal effects were found on haematological and clinical biochemical parameters monitored longitudinally during the dietary study (section S2, Table S5), but clear differences between diet groups were observed for total Se, selenospecies and VitE levels in plasma and tissues, which are important factors for the antioxidant status and the immunological response capacity of finisher pigs as proven with the current LPS challenge and in previous studies [31, 45, 46].

### Influence of Diet on the Antioxidant Status of Pigs

The significantly higher levels of total Se and selenospecies in plasma of pigs fed SeMet-supplemented diets suggest an improvement in antioxidant status throughout the 80-day trial compared to pigs fed NaSe-supplemented and Control diets. The differences observed in total Se plasma concentrations (Fig. 1, Table S6) are in good agreement with previous dietary studies reporting higher plasma Se levels in sows and piglets fed SeMet diets [31, 32] and in other mammal species fed diets enriched with organic Se sources [47, 48]. This effect of dietary Se-source was also evident on the selenospecies levels throughout the trial period (Fig. 2, Table S7). Plasma SeMet, GPx3, SelP and SeAlb concentrations in pigs fed SeMet-0.44 and SeMix diets were generally higher than those determined in pigs fed the sole Se source diets complying with the current EU-legislation (NaSe-0.40, SeMet-0.19) and comparable with those in pigs receiving the highest total Se in the feed (NaSe-0.65; above EU-legislated limits). More notably, the SeMet-0.44 diet led to significantly higher plasma GPx3 levels already from day 16 into the trial. This improvement in levels of Se-containing proteins by supplementing the feed with SeMet compared with NaSe at a dietary level of 0.40 mg Se/kg supports the theory of higher bioavailability of organic Se compared to inorganic Se as previously described in [49].

The dietary effect on plasma Se levels is also reflected in the observed differences in tissue Se accumulation, particularly in muscle tissues where a reinforced effect of Se source was shown. As seen in other dietary porcine studies [33, 50], the extent of tissue accumulation increased with higher concentration in the feed, yet our results also prove that the source of Se used yields an even stronger effect (Fig. 3, Tables S10). Significantly higher total Se concentrations were measured in the muscle and brain tissues from pigs fed any of the diets containing SeMet than in tissues of pigs fed NaSe and Control diets. Interestingly, the combined Se source diets (SeMix-1, SeMix-2) resulted in higher total Se concentrations in all examined tissues, including liver and kidney, than with the other diets supplemented as approved by the EU legislation (i.e. NaSe-0.40 and SeMet-0.19). This combined dietary source effect on Se accumulation in tissues agrees with the incorporation of not metabolized SeMet into body proteins [11, 26] and the longer whole-body half-life reported for SeMet compared with inorganic Se [51] in addition to the synthesis of selenoproteins from selenite. The subsequent release of stored SeMet in tissues together with the ingested selenite via these mixed source diets support an optimal synthesis of selenoproteins. Thus, and as suggested by [32], the pigs fed SeMet, alone or in combination with NaSe, are expected to maintain higher activity of selenoenzymes during periods of higher antioxidant demand, e.g. after the weaning process when piglets often consume less feed (commonly known as post-weaning growth check) and can be more susceptible to infection due to increased stress levels [52].

The antioxidant status of the pigs was also influenced by the VitE levels in plasma and tissues. By the end of the trial, a two-fold higher VitE concentration in the feed resulted in significantly higher VitE plasma and tissues levels in pigs fed a non-Se-supplemented (Control-2 *vs. *Control-1) as well as in pigs fed diets with the same Se-supplement (SeMix-2 *vs.* SeMix-1) (Figs. 1 and 3, Tables S6 and S9). This strong positive correlation between VitE feed concentration and its accumulation in plasma and tissues is in agreement with the feed concentration effect previously reported for VitE levels in plasma, liver and muscles of grower pigs fed non-Se-enriched diets containing 80 or 130 mg VitE/kg compared to diets with ~ 20 mg VitE/kg [53, 54], in serum, colostrum and milk of sows fed Se-enriched diets (0.35 mg/kg) containing 105 mg VitE/kg compared with 45 mg VitE/kg enriched diets [50], and in serum, liver and adipose tissue of weaned pigs fed dietary levels of 0.3 mg Se/kg and between ~ 13 and 130 mg VitE/kg [37].

Furthermore, when supplemented together with Se, the VitE levels increased in plasma and most tissues. Pigs fed Se-supplemented diets containing the same ~ 50 mg VitE/kg as in the Control-2 diet (i.e. NaSe-0.65, SeMet-0.19, SeMet-0.44, SeMix-1) showed higher VitE levels (Figs. 1 and 3), and increasing the Se concentration in the feed either as NaSe or SeMet (i.e. NaSe-0.65 *vs.* NaSe-0.40 and SeMet-0.44 *vs.* SeMet-0.19) resulted in further enhancement of the VitE levels. Notably, when comparing pigs fed the same VitE and Se concentrations in the feed (i.e. ~ 50 and ~ 0.4 mg/kg, respectively), pigs fed any of the SeMet-supplemented diets accumulated by the end of the trial higher average concentration of VitE in plasma (SeMet-0.44) and tissues (SeMet-0.44, SeMix-1) than pigs fed the low NaSe-enriched diet (NaSe-0.40), confirming a positive Se source effect on the VitE status. These results suggest that there is a VitE-Se synergistic effect against oxidative stress as already reported for other mammals [55] and fish [56]. Here, the longitudinal dietary study showed that supplementing diets with SeMet (i.e. improving the overall Se status) and ≥ 50 mg/kg VitE (i.e. sufficient VitE for promoting a positive accumulation interaction between Se and VitE) resulted in a beneficial effect on the antioxidant status of finisher pigs.

### Antioxidant Response Following LPS Exposure

Injecting the LPS intravenously half-way through the feeding trial provoked a similar pyrogenic response within the first 3 h in all pigs across all diet groups and increased the pigs’ heart rate, as shown in an earlier study [57]. The vital parameters approached initial values and all pigs resumed normal behaviour, including feed and water intake, 8 h after injection, suggesting that the injected LPS (2 µg/kg BW) was neutralized rather rapidly in pigs of all diet groups after administration, likely due to detoxification by the enzyme acyloxyacyl hydrolase [58]. This response agrees with results from previous feeding trials of gilts and sows injected with 1.5–2 µg LPS/kg BW intravenously [31, 59]. The significantly higher LDH plasma activity in pigs fed the Control-1 diet compared with the other diets groups indicates that a higher level of oxidative stress occurred in the pigs fed this non-Se- and non-VitE-supplemented diet [60]. This diet effect is supported by the differences in total Se and selenospecies as well as VitE levels determined in plasma prior to the LPS injection, i.e. pigs had different initial antioxidant statuses (days 49–56 into the trial; Figs. 1 and 2).

The LPS-induced oxidative stress led to a direct response of the Se metabolism but to a different extent depending on the diet fed to the pigs. The significant rise of inorganic Se in plasma of all pigs between 5 and 12 h after the injection (Fig. 4) accompanied by a continuous increase of GPx3 and SelP levels in plasma, especially after 12 h (Fig. 5), suggests a boosting of readily available SeCys for conversion into H_2_Se and subsequent synthesis of selenoproteins [8, 26]. This selenoprotein upregulation, supported by protein degradation and suppression of synthesis of non-vital proteins [61], demonstrates a rapid response to the production of different ROS and pro-inflammatory mediators during oxidative stress. This upregulation was particularly evident in pigs fed the SeMet-0.44 diet, and to a similar extend in those fed SeMix-1 and SeMix-2 diets. These pigs maintained the highest plasma levels of total Se, GPx3 and SelP concentrations not just during the 48-h response period after the LPS injection, but also throughout the dietary trial (Fig. 2), thus demonstrating that an improved initial Se status had a positive effect on the antioxidant capacity of the pigs. Likewise, the increase of plasma SeAlb levels starting 12 h after the LPS injection can be linked to the significant decrease observed in SeMet levels between 12–24 h, which suggests a relocation of available SeMet into plasma albumin. Free SeMet in SeMet fed animals may have originated from the constant dietary intake and/or release from post-LPS protein degradation [61], or from maternal and plant/raw material derived SeMet in selenite fed animals as suggested for piglets from selenite-fed sows [32]. However, the high SeMet levels observed in NaSe-0.65 fed pigs soon after the LPS injection, without an associated change in SeAlb levels, might be due to additional oxidative stress caused by excess selenite in these pigs (i.e. highest initial inorganic Se plasma levels; Fig. 2). Metabolising the rather oxidizing selenite can induce ROS production [62], possibly causing a greater protein degradation in these selenite-fed pigs, followed by SeMet release, compared to those fed SeMet-supplemented diets. Overall, these results are in agreement with previous dietary studies that found SeMet-supplemented diets fed to pigs [32, 43, 50] and other species such as fish [63] to be more efficient against oxidative stress than selenite-enriched diets due to retention of Se in tissues and more stable total Se levels in plasma.

Furthermore, the upregulation evaluated as the rise of plasma GPx3 concentrations from initial levels (Fig. 5) was more significant in pigs fed the SeMet-0.44 and SeMix diets (i.e. 8 h after the LPS injection) compared with the 24-h response time observed in pigs receiving the non-Se-supplemented diets, the selenite-supplemented diets at or above the EU-legislated feed limits or the SeMet-supplemented diet within EU-legislation (no significant differences between these five diet groups at 8, 12 and 24 h; Table S8). This difference in response can be explained by the Se status attained by each diet group prior to the LPS injection (Figs. 1 and 2). Mou et al. [46] reported a lower anti-inflammatory response in weaning pigs from sows receiving a selenite-enriched diet than those fed a hydroxy-SeMet diet (both at 0.3 mg Se/kg), which showed a significant increase in GPx3 activity already 4 h after a LPS challenge. This faster response was correlated to the enhanced SelP levels observed in piglets’ plasma during lactation. In our study, the SeMet-supplemented diets (SeMet-0.44, SeMix-1 or SeMix-2) resulted in higher levels of GPx3 and SelP in plasma than the other diets, improving the Se-based antioxidant response capacity in the pigs.

Finally, some indications of enhanced protection against oxidative stress were observed in tissues of LPS-exposed pigs fed SeMet-supplemented diets compared to those fed selenite- and non-Se-supplemented diets (Figs. 6 and S6). Dietary Se deficiency with or without low VitE intake (Control-1, Control-2) resulted in a higher number of lesions in the heart muscle, whereas the SeMet and medium VitE concentration diets (SeMet-0.44, SeMix-1) resulted in generally milder and fewer lesions in the *M*. *longissimus dorsi* than the other diets. At the same time, these SeMet-fed pigs, especially the SeMet-0.44 group, showed significantly improved Se and VitE tissue status than all other pigs (Fig. 3). These results together demonstrate a beneficial effect of the dietary SeMet in LPS-exposed pigs, confirming the key role of tissue Se reserves for maintaining the selenoprotein synthesis during an antioxidant response. Therefore, through enhanced SeMet body reserves and continuous organic Se feed supplement, the pigs fed SeMet-diets were more robust towards inflammatory insults and oxidative injuries, as seen in [31]. Ultimately, enhanced Se levels in pork products can contribute to an adequate Se intake in humans living in Se deficient soil regions, such as New Zealand, China or the Balkan and Scandinavian countries [64].

## Conclusions

To the authors’ knowledge, this is the first study reporting Se speciation, i.e. inorganic Se, SeCys, SeMet, GPx3, SelP and SeAlb distribution in plasma, along with total Se and VitE in plasma and various tissues of finisher pigs. The results are important from a clinical point of view since pigs are prone to conditions linked to Se and/or VitE deficiency during periods of rapid growth or exposure to exogenous sources of oxidative stress. Supplying Se in an organic form resulted in a beneficial effect in animal health with improved Se tissue accumulation and a most robust antioxidant status and, even when combining it with an inorganic Se source, an improvement in antioxidant status towards LPS-induced oxidative stress was observed. Therefore, finisher pigs fed diets comparable to the SeMix diets (i.e. ~ 0.2 mg Se/kg as SeMet, ~ 0.2 mg Se/kg as sodium selenite) at the required daily feed intake will likely be less susceptible to MHD or NMD conditions. By doubling the allowed organic Se concentration in the feed (i.e. up to 0.44 mg Se/kg), the Se status was additionally improved and the accumulation of Se in all tissues increased up to sixfold without compromising the animal’s health due to overload. This improvement in Se body levels also affected the VitE levels positively, thereby increasing the overall antioxidant status of the pigs. The benefits of higher accumulation and improved antioxidant properties via SeMet- and VitE-enriched diets such as SeMet-0.44 may help strengthening the immune status and resilience of todays’ high-yielding genetic lines. This study hence highlights the need for revising the dietary limits of organic Se in swine rations to improve the levels of bioavailable Se in feed-efficient pigs.

## Supplementary Information

Below is the link to the electronic supplementary material.

Supplementary file 1 contains additional information on the pigs’ body weight, feed intake, and visits to the feeder during the dietary study; haematology and clinical biochemistry for the dietary and LPS challenge studies (methodology, results, significance tests); clinical examinations for the LPS challenge study; total Se, VitE and Se speciation in plasma for the dietary and LPS challenge studies (representative chromatograms, average concentrations, significance tests); tissues *post-mortem* (average concentrations, significance tests, histopathology scoring). (PDF 1.30 MB)

## Data Availability

All datasets generated and analysed during this study are included in this published article and its supplementary information file, with exception of the raw clinical examination measurements that can be available from the corresponding author on reasonable request.

## Abbreviations

BHT: Butylated hydroxytoluene
BPM: Beats per minute
BW: Body weight
EU: European Union
GPx: Glutathione peroxidase
H_2_Se: Selenide
HFBA: Heptafluorobutyric acid
HPLC: High-performance liquid chromatography
ICP-MS: Inductively coupled plasma mass spectrometry
LPS: Lipopolysaccharide
MHD: Mulberry heart disease
*N*: Number of animals
*n*: Number of measurements
NaSe: Sodium selenite
NMD: Nutritional muscular dystrophy
ROS: Reactive oxygen species
RP-HPLC: Reversed phase high-performance liquid chromatography
RSD: Relative standard deviation
Se: Selenium
SeAlb: Selenoalbumin
SeCys: Selenocysteine
SD: Standard deviation
SelP: Selenoprotein P
SeMet: Selenomethionine
VitE: Vitamin E
