# Supplementary material for: Selenium Speciation Analysis Reveals Improved Antioxidant Status in Finisher Pigs Fed l-Selenomethionine, Alone or Combined with Sodium Selenite, and Vitamin E
Source: Biol Trace Elem Res. 2022 Dec 29;201(9):4400–18. doi: 10.1007/s12011-022-03516-9 (PMC10350441; doi:10.1007/s12011-022-03516-9)
Supplement: Supplementary file 1 — contains additional information on the pigs’ body weight, feed intake, and visits to the feeder during the dietary study; haematology and clinical biochemistry for the dietary and LPS challenge studies (methodology, results, significance tests); clinical examinations for the LPS challenge study; total Se, VitE and Se speciation in plasma for the dietary and LPS challenge studies (representative chromatograms, average concentrations, significance tests); tissues post-mortem (average concentrations, significance tests, histopathology scoring). (PDF 1.30 MB) [file 12011_2022_3516_MOESM1_ESM.pdf]

Supplementary Information for

Selenium Speciation Analysis Reveals Improved Antioxidant Status in Finisher Pigs Fed L-Selenomethionine, Alone or Combined With Sodium Selenite, and Vitamin E

Estela REINOSO-MASET[1,*], Michaela FALK[2,3,], Aksel BERNHOFT[4], Cecilie ERSDAL[3], Tore FRAMSTAD[5], Herbert FUHRMANN[6], Brit SALBU[1], Marianne OROPEZA-MOE[3]

[1] Centre for Environmental Radioactivity CoE, Faculty of Environmental Sciences and Natural Resource Management, Norwegian University of Life Sciences, Elizabeth Stephansens vei 31, 1433 Aas, Norway

[2] Norwegian Veterinary Institute, Svebastadveien 112, 4325 Sandnes, Norway

[3] Department of Production Animal Clinical Sciences (PRODMED), Faculty of Veterinary Medicine, Norwegian University of Life Sciences, Svebastadveien 112, 4325 Sandnes, Norway

[4] Norwegian Veterinary Institute, Elizabeth Stephansens vei 1, 1433 Aas, Norway

[5] Department of Production Animal Clinical Sciences (PRODMED), Faculty of Veterinary Medicine, Norwegian University of Life Sciences, Elizabeth Stephansens vei 15, 1433 Aas, Norway

[6] Institute of Physiological Chemistry, Faculty of Veterinary Medicine, University of Leipzig, 04103 Leipzig, Germany

[*] *Corresponding author:* estela.reinoso.maset@nmbu.no

Biological Trace Element Research
November 2022

Table of contents

S1. BODY WEIGHT, FEED INTAKE, VISITS TO FEEDER 2
Body weight gain 2
Weekly pen averages 5
S2. HAEMATOLOGY AND CLINICAL BIOCHEMISTRY 8
Analytical methodology 8
Blood parameters results 8
Dietary study 8
LPS challenge study 9
S3. CLINICAL EXAMINATIONS 11
S4. TOTAL SELENIUM, VITAMIN E, AND SELENIUM SPECIATION 12
Analytical methodology 12
Results 13
Plasma concentrations during the dietary study 13
Plasma concentrations during the LPS challenge study 16
S5. TISSUES POST-MORTEM 20
Results 20
Selenium and vitamin E concentrations 20
Histopathology lesion scoring 21
REFERENCES 21

S1. BODY WEIGHT, FEED INTAKE, VISITS TO FEEDER

Body weight gain

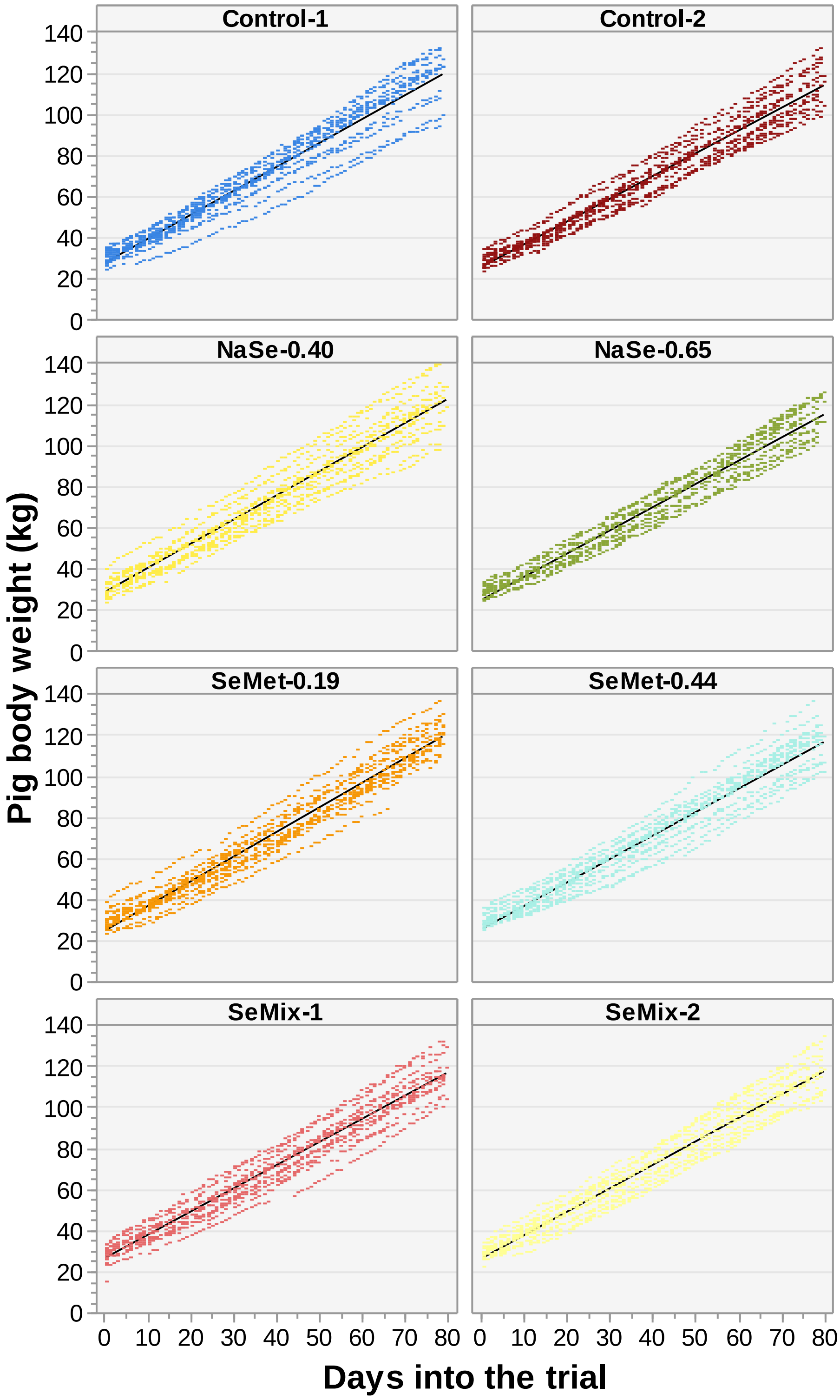

Figure S1. Animal body weight (in kg) recorded during the trial (in days) for each pig individual (N = 16 per diet) fed non-Se-supplemented (Control) or NaSe and/or SeMet enriched diets (Table 1). Equations of the linear regressions (black lines) for each diet group are shown in Table S2.

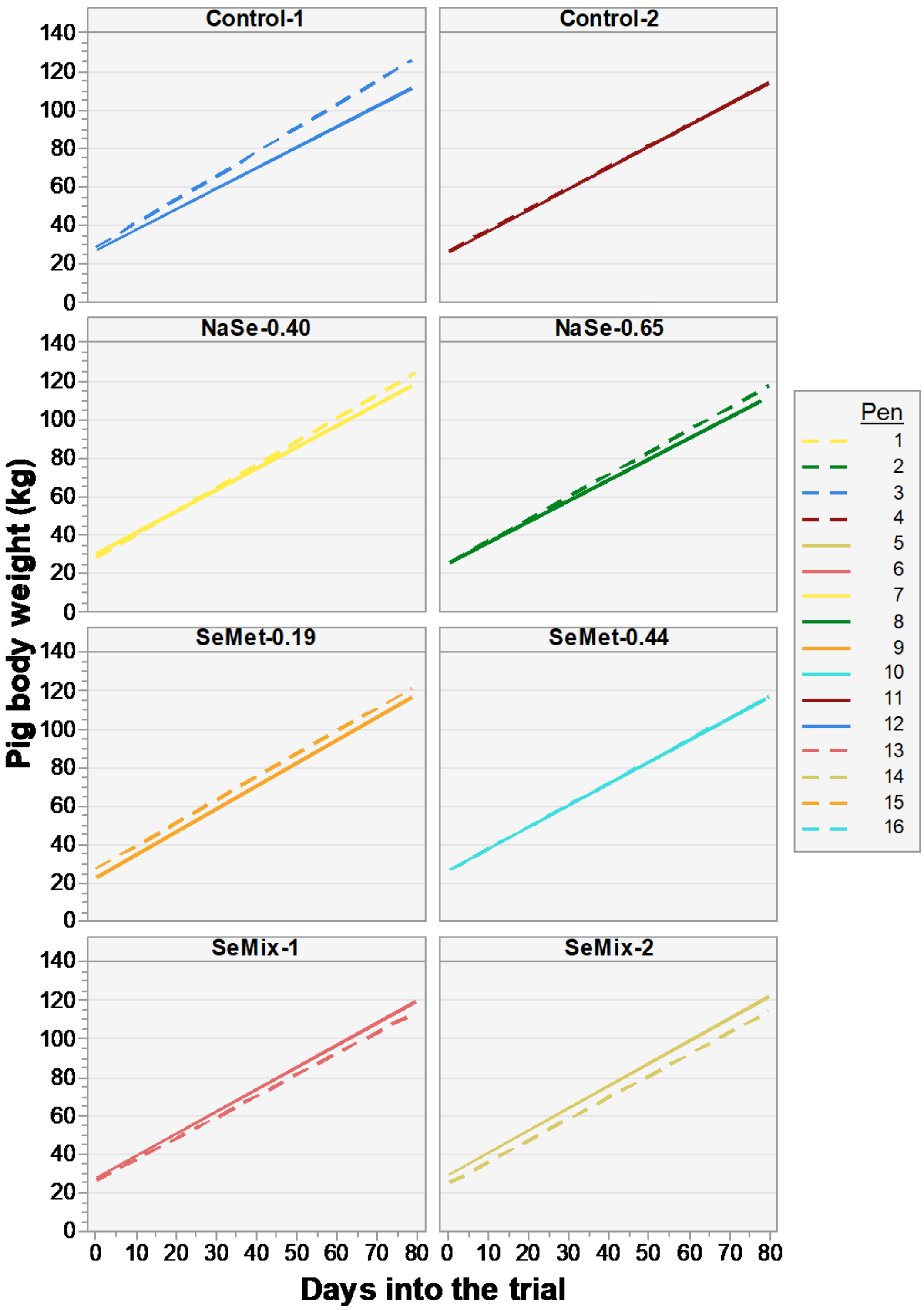

Figure S2. Linear regression lines of body weight gain (in kg) over time (in days) for pigs within a pen fed non-Se-supplemented (Control) or NaSe and/or SeMet enriched diets (Table 1). Pens numbered 5, 6, 7, 8, 9, 10, 11, 12 hosted 7 pigs (full lines) and pens numbered 1, 2, 3, 4, 13, 14, 15, 16 hosted 9 pigs (dashed lines). *NOTE:* No clear differences in BW gain rate were observed between pens, except for pens 3 and 12 of Control-1 diet group. The average BW of pigs hosted in these pens were already ca. 3 kg different at the beginning of the trial (Table S1), which may have resulted in lower weekly BW averages throughout the whole trial (Table S2) [1].

Table S1. Average body weight (1 standard deviation (SD), *n* = number of measurements) at the start (day 0) and end of the trial (day 78-80) for pigs fed non-Se-supplemented (Control) and NaSe and/or SeMet enriched diets (Table 1; *N* = 16 per diet). Averages of BW that do not share a grouping letter are significantly different (one-way ANOVA, Tukey pairwise, 99.82% confidence level). The average weight gain (in kg per day) for each diet is given by the slope of the linear regression of the BW recorded during the feeding trial for all pigs within a diet group (Figure S1).

	Body weight, kg									Body weight *vs.* time	
		at trial start (day 0)				at trial end (day 78-80)[a]					
Diet	**Pen**	***n***	**Avg.**	**SD**	**grouping[b]**	***n***	**Avg.**	**SD**	**grouping[b]**	**Linear regression**	**R^2**
Control-1		16	30.1	3.1	A	14	121.0	13.2	A	y = 27.7 + 1.16x	0.923
	3	9	31.4	2.6	a	8	125.1	8.3	a		
	12	7	28.4	3.0	a	6	115.5	17.2	a		
Control-2		16	28.2	3.2	A	15	114.7	10.4	A	y = 26.2 + 1.10x	0.941
	4	9	28.1	2.3	a	9	113.5	9.7	a		
	11	7	28.3	4.2	a	6	116.6	12.1	a		
NaSe-0.40		16	29.8	4.2	A	15	118.8	12.9	A	y = 28.6 + 1.17x	0.917
	1	9	29.5	3.2	a	8	122.9	10.9	a		
	7	7	30.1	5.6	a	7	114.2	14.3	a		
NaSe-0.65		15	28.3	2.8	A	14	114.8	8.7	A	y = 25.5 + 1.12x	0.951
	2	9	28.5	2.6	a	9	117.3	7.4	a		
	8	6[c]	27.9	3.2	a	5	110.2	9.7	a		
SeMet-0.19		16	29.0	4.0	A	14	121.5	7.8	A	y = 25.4 + 1.19x	0.941
	9	7	27.0	2.3	a	6	120.7	5.8	a		
	15	9	30.5	4.5	a	8	122.0	9.4	a		
SeMet-0.44		16	30.5	3.8	A	15	116.6	8.6	A	y = 26.7 + 1.13x	0.932
	10	7	30.1	3.1	a	7	117.2	9.5	a		
	16	9	30.8	4.4	a	8	116.1	8.4	a		
SeMix-1		16	28.4	4.6	A	15	117.1	8.9	A	y = 26.8 + 1.13x	0.931
	6	7	28.1	6.0	a	7	118.6	6.7	a		
	13	9	28.6	3.5	a	9	116.0	10.5	a		
SeMix-2		16	28.9	2.7	A	16	117.6	9.8	A	y = 27.2 + 1.13x	0.939
	5	7	30.2	2.6	a	6	121.2	10.9	a		
	14	9	27.8	2.5	a	9	115.2	8.8	a		
All diets		*127*	*29.1*	*3.6*		*118*	*117.7*	*10.2*		*y = 26.8 + 1.14x*	*0.930*

[a] Average of the last recorded weight fir each pig between day 78 and 80.
[b] Uppercase grouping letters for comparison between diet group averages; lowercase letters for comparison between the average BW of pigs hosted in the 7- and 9-pig pens.
[c] One pig not weighed out automatically on day 0.

Weekly pen averages

Table S2. Weekly pen average of body weight (1 standard deviation (SD), *n* = number of measurements) for pigs fed non-Se-supplemented (Control) and NaSe and/or SeMet enriched diets (Table 1; *N* = 16 per diet). Averages are given with the same number of decimals recorded by the automatic feeder. Pens numbered 5-12 hosted 7 pigs and pens numbered 1-4 and 13-16 hosted 9 pigs.

			Weekly body weight average, kg										
Diet	**Pen**	**Week**	**1**	**2**	**3**	**4**	**5**	**6**	**7**	**8**	**9**	**10**	**11-12**
Control-1	3	*Avg.*	34.5	40.9	48.4	57.1	65.9	74.5	83.2	91.6	101.2	110.6	121.3
		SD	2.9	3.1	3.5	3.6	4.0	4.4	4.5	5.3	6.3	6.6	7.4
		n	54	55	58	58	51	48	47	52	48	48	63
	12	*Avg.*	31.9	37.4	44.1	52.0	59.3	66.3	74.6	81.8	89.7	96.8	108.2
		SD	3.8	4.8	6.0	6.7	7.1	7.9	9.3	9.6	9.8	10.2	12.3
		n	44	48	40	40	44	43	44	43	44	37	48
Control-2	4	*Avg.*	31.4	37.3	44.2	52.0	60.1	67.3	76.5	83.4	91.0	98.8	108.7
		SD	2.6	2.4	3.0	3.4	3.9	4.2	4.6	5.5	6.1	7.6	9.6
		n	58	58	56	54	61	51	53	45	54	58	66
	11	*Avg.*	31.5	37.1	44.3	51.9	58.1	66.1	74.1	82.5	89.5	99.1	109.9
		SD	4.6	5.1	5.8	7.0	7.3	8.3	8.7	9.2	9.0	9.8	10.9
		n	42	37	48	40	43	40	46	45	42	47	60
NaSe-0.40	1	*Avg.*	33.3	40.5	47.8	55.5	64.8	72.8	81.7	89.8	99.0	108.0	119.0
		SD	4.0	4.3	5.3	6.0	6.7	7.0	7.6	8.2	8.8	9.4	9.9
		n	58	61	55	52	52	48	52	51	56	51	73
	7	*Avg.*	33.9	41.1	48.1	56.1	64.2	72.2	80.6	89.0	96.6	103.6	110.7
		SD	6.1	6.6	6.6	6.9	7.7	9.3	9.3	10.8	12.2	12.0	13.9
		n	42	41	39	44	47	45	43	41	37	37	52
NaSe-0.65	2	*Avg.*	31.5	37.9	44.7	52.3	60.6	68.1	76.1	82.7	92.9	103.5	113.0
		SD	3.4	4.1	4.4	4.2	5.2	5.9	5.7	5.5	6.5	6.6	7.6
		n	51	53	54	53	60	59	59	52	60	57	77
	8	*Avg.*	30.6	36.5	43.3	49.2	56.5	64.5	72.5	83.0	89.5	96.7	106.2
		SD	3.5	3.9	4.3	5.6	6.3	7.0	7.4	7.3	7.3	8.4	8.9
		n	36	30	26	28	26	24	25	21	34	42	49
SeMet-0.19	9	*Avg.*	30.2	36.2	42.7	50.1	57.2	66.1	74.9	83.3	92.9	102.0	115.1
		SD	3.1	3.9	4.3	4.7	5.0	5.5	5.7	6.8	6.3	7.5	5.9
		n	45	47	43	44	47	46	44	45	44	40	53
	15	*Avg.*	34.3	39.6	47.2	53.8	62.8	71.5	80.1	89.3	96.8	107.2	117.5
		SD	5.4	5.5	6.2	6.2	7.2	7.7	8.1	8.4	8.8	9.3	9.1
		n	58	65	52	52	53	52	55	51	54	55	67
SeMet-0.44	10	*Avg.*	32.9	38.6	45.8	52.9	60.2	67.5	76.0	85.1	93.5	103.2	112.5
		SD	3.5	4.1	4.6	5.4	6.3	6.6	7.1	6.9	7.8	8.4	8.9
		n	46	49	48	49	45	44	47	47	45	45	59
	16	*Avg.*	32.5	38.0	45.1	53.6	60.1	68.8	77.6	84.3	93.8	101.4	111.8
		SD	4.2	4.3	5.0	6.2	7.4	7.9	8.8	9.9	9.7	9.8	10.1
		n	55	58	62	57	55	59	56	46	55	57	83
SeMix-1	6	*Avg.*	33.2	40.3	46.2	52.9	62.0	70.3	78.5	88.0	96.1	103.7	113.6
		SD	4.9	3.6	4.0	4.9	4.5	4.9	5.5	5.0	5.8	6.0	6.8
		n	41	33	31	37	40	33	41	42	41	43	59
	13	*Avg.*	31.4	37.8	44.1	51.5	60.1	66.9	74.2	82.8	90.6	99.2	110.4
		SD	3.7	4.8	6.1	7.4	8.1	8.5	8.5	9.9	9.9	10.6	10.7
		n	58	53	51	54	53	53	52	53	53	55	66
SeMix-2	5	*Avg.*	33.7	41.3	49.3	56.6	64.8	72.9	80.3	89.5	98.5	105.3	116.5
		SD	3.9	4.6	4.6	4.8	5.6	6.2	7.0	7.9	8.2	8.3	8.6
		n	46	49	47	46	45	38	44	48	44	37	55
	14	*Avg.*	31.4	37.0	43.3	50.5	57.3	65.3	82.3	82.0	92.0	99.6	110.0
		SD	3.2	4.3	5.0	5.7	5.5	5.7	6.5	6.4	7.4	7.7	8.7
		n	57	52	51	53	49	51	53	50	55	57	75
All diets		*Avg.*	32.4	38.6	45.1	52.6	61.0	68.9	77.3	85.6	94.0	102.4	112.9
		SD	4.2	4.7	5.2	6.1	6.8	7.4	7.8	8.5	8.9	9.5	10.2
		n	791	782	660	861	771	734	757	731	766	766	1004

Table S3. Weekly pen average of feed intake (1 standard deviation (SD), *n* = number of measurements) for pigs fed non-Se-supplemented (Control) and NaSe and/or SeMet enriched diets (Table 1; *N* = 16 per diet). Averages are given with the same number of decimals recorded by the automatic feeder. Pens numbered 5-12 hosted 7 pigs and pens numbered 1-4 and 13-16 hosted 9 pigs.

			**Weekly feed intake average, kg**										
Diet	**Pen**	**Week**	**1**	**2**	**3**	**4**	**5**	**6**	**7**	**8**	**9**	**10**	**11-12**
Control-1	3	*Avg.*	1.557	1.973	2.254	2.483	2.723	3.096	3.406	3.398	3.736	3.892	3.663
		SD	0.557	0.308	0.392	0.476	0.440	0.408	0.473	0.705	0.549	0.699	1.135
		n	54	55	58	58	51	48	47	52	48	48	63
	12	*Avg.*	1.437	1.732	2.124	2.332	2.467	2.798	2.942	2.993	3.159	3.192	3.449
		SD	0.608	0.380	0.473	0.506	0.562	0.623	0.704	0.709	0.714	0.809	0.962
		n	44	48	40	40	44	43	44	43	44	37	48
Control-2	4	*Avg.*	1.436	1.689	1.979	2.291	2.532	2.813	3.109	2.858	3.305	3.460	3.120
		SD	0.520	0.332	0.361	0.327	0.379	0.423	0.528	0.637	0.518	0.673	1.046
		n	58	58	56	54	61	51	53	45	54	58	66
	11	*Avg.*	1.065	1.375	1.677	1.998	2.302	2.445	2.709	2.986	3.034	3.082	3.111
		SD	0.541	0.396	0.428	0.491	0.460	0.543	0.431	0.531	0.544	0.578	1.115
		n	42	37	48	40	43	40	46	45	42	47	60
NaSe-0.40	1	*Avg.*	1.342	1.889	2.108	2.446	2.713	2.913	3.079	3.176	3.447	3.712	3.704
		SD	0.568	0.347	0.479	0.434	0.481	0.512	0.634	0.713	0.674	0.793	1.114
		n	58	61	55	52	52	48	52	51	56	51	73
	7	*Avg.*	2.038	1.874	2.054	2.261	2.483	2.711	2.897	2.920	2.953	3.206	3.181
		SD	1.122	0.409	0.320	0.502	0.464	0.710	0.665	0.722	0.852	0.839	0.783
		n	42	41	39	44	47	45	43	41	37	37	52
NaSe-0.65	2	*Avg.*	1.275	1.810	2.080	2.563	2.553	2.797	3.132	3.132	3.494	3.726	3.522
		SD	0.623	0.382	0.387	0.384	0.512	0.642	0.462	0.578	0.517	0.581	1.167
		n	51	53	54	53	60	59	59	52	60	57	77
	8	*Avg.*	1.236	1.721	1.980	2.183	2.312	2.720	2.888	3.220	3.130	3.290	3.509
		SD	0.513	0.314	0.483	0.303	0.275	0.448	0.455	0.388	0.638	0.708	0.616
		n	36	30	26	28	26	24	25	21	34	42	49
SeMet-0.19	9	*Avg.*	1.399	1.729	1.896	2.140	2.462	2.772	2.970	3.184	3.475	3.719	3.746
		SD	0.428	0.287	0.382	0.359	0.385	0.377	0.370	0.534	0.496	0.552	0.986
		n	45	47	43	44	47	46	44	45	44	40	53
	15	*Avg.*	1.446	1.715	2.172	2.204	2.518	2.882	3.083	3.292	3.415	3.778	3.842
		SD	0.494	0.354	0.326	0.441	0.378	0.468	0.549	0.592	0.565	0.611	0.877
		n	58	65	52	52	53	52	55	51	54	55	67
SeMet-0.44	10	*Avg.*	1.263	1.830	1.993	2.215	2.498	2.691	2.892	3.144	3.252	3.376	3.448
		SD	0.549	0.324	0.374	0.341	0.382	0.387	0.404	0.397	0.470	0.543	0.889
		n	46	49	48	49	45	44	47	47	45	45	59
	16	*Avg.*	1.198	1.665	1.959	2.300	2.425	2.763	3.007	3.045	3.312	3.522	3.474
		SD	0.543	0.297	0.340	0.461	0.434	0.518	0.531	0.519	0.476	0.554	0.858
		n	55	58	62	57	55	59	56	46	55	57	83
SeMix-1	6	*Avg.*	1.390	1.655	1.894	2.318	2.363	2.589	2.811	3.175	3.359	3.283	3.042
		SD	0.603	0.399	0.341	0.302	0.356	0.349	0.346	0.411	0.454	0.653	0.906
		n	41	33	31	37	40	33	41	42	41	43	59
	13	*Avg.*	1.306	1.859	2.115	2.271	2.489	2.682	2.984	3.088	3.316	3.462	3.384
		SD	0.453	0.511	0.560	0.695	0.569	0.621	0.583	0.764	0.552	0.614	0.946
		n	58	53	51	54	53	53	52	53	53	55	66
SeMix-2	5	*Avg.*	1.456	2.033	2.190	2.424	2.590	2.604	2.917	3.307	3.391	3.377	3.158
		SD	0.670	0.334	0.398	0.493	0.442	0.643	0.485	0.544	0.503	0.688	1.016
		n	46	49	47	46	45	38	44	48	44	37	55
	14	*Avg.*	1.385	1.631	1.990	2.173	2.328	2.596	2.872	2.880	3.238	3.443	3.246
		SD	0.412	0.432	0.329	0.334	0.321	0.285	0.557	0.572	0.432	0.548	1.062
		n	57	52	51	53	49	51	53	50	55	57	75
All diets		*Avg.*	1.388	1.771	2.009	2.288	2.495	2.752	2.993	3.114	3.328	3.487	3.418
		SD	0.611	0.390	0.413	0.458	0.451	0.530	0.529	0.616	0.584	0.684	1.009
		n	791	782	660	861	771	734	757	731	766	766	1004

Table S4. Weekly pen average of visits to the feeder per day (1 standard deviation (SD), *n* = number of measurements) for pigs fed non-Se-supplemented (Control) and NaSe and/or SeMet enriched diets (Table 1; *N* = 16 per diet). Pens numbered 5-12 hosted 7 pigs and pens numbered 1-4 and 13-16 hosted 9 pigs.

			**Weekly feeder visits average, visits/day**										
Diet	**Pen**	**Week**	**1**	**2**	**3**	**4**	**5**	**6**	**7**	**8**	**9**	**10**	**11-12**
Control-1	3	*Avg.*	10	9	11	13	12	10	10	9	9	9	9
		SD	4	2	3	4	4	3	3	3	4	3	4
		n	54	55	58	58	51	48	47	52	48	48	63
	12	*Avg.*	13	11	11	12	9	9	9	9	9	8	7
		SD	4	3	4	5	4	3	3	3	3	3	3
		n	44	48	40	40	44	43	44	43	44	37	48
Control-2	4	*Avg.*	15	11	11	11	9	9	9	8	7	8	6
		SD	9	3	3	4	3	3	3	3	2	2	2
		n	58	58	56	54	61	51	53	45	54	58	66
	11	*Avg.*	12	10	10	11	13	14	14	13	11	10	8
		SD	5	3	4	4	5	5	5	4	4	3	4
		n	42	37	48	40	43	40	46	45	42	47	60
NaSe-0.40	1	*Avg.*	14	10	11	11	9	9	8	8	8	8	7
		SD	6	3	3	4	2	2	2	2	2	2	3
		n	58	61	55	52	52	48	52	51	56	51	73
	7	*Avg.*	13	11	11	11	11	11	13	13	12	12	11
		SD	4	3	3	3	5	5	5	4	4	4	4
		n	42	41	39	44	47	45	43	41	37	37	52
NaSe-0.65	2	*Avg.*	12	7	8	8	8	8	7	7	8	7	7
		SD	6	2	2	2	3	2	2	3	3	3	3
		n	51	53	54	53	60	59	59	52	60	57	77
	8	*Avg.*	12	8	9	9	9	9	9	11	10	10	9
		SD	6	1	3	2	2	2	2	2	3	3	3
		n	36	30	26	28	26	24	25	21	34	42	49
SeMet-0.19	9	*Avg.*	13	12	13	13	12	12	10	10	10	9	8
		SD	4	4	4	4	4	4	2	2	3	3	3
		n	45	47	43	44	47	46	44	45	44	40	53
	15	*Avg.*	11	11	12	12	9	9	8	8	9	8	8
		SD	4	3	3	4	3	3	3	3	3	3	3
		n	58	65	52	52	53	52	55	51	54	55	67
SeMet-0.44	10	*Avg.*	11	10	10	10	9	9	9	9	8	7	7
		SD	4	2	3	3	3	3	3	3	3	2	3
		n	46	49	48	49	45	44	47	47	45	45	59
	16	*Avg.*	12	9	10	9	9	10	11	10	10	10	9
		SD	5	2	2	3	2	3	4	4	3	3	4
		n	55	58	62	57	55	59	56	46	55	57	83
SeMix-1	6	*Avg.*	11	9	10	12	10	8	9	8	9	8	7
		SD	3	4	3	3	4	2	3	2	3	3	3
		n	41	33	31	37	40	33	41	42	41	43	59
	13	*Avg.*	12	10	10	10	9	9	9	8	9	8	7
		SD	5	4	4	4	3	4	4	4	4	3	3
		n	58	53	51	54	53	53	52	53	53	55	66
SeMix-2	5	*Avg.*	10	8	8	8	8	7	7	8	8	8	6
		SD	4	2	3	2	2	2	3	2	2	2	3
		n	46	49	47	46	45	38	44	48	44	37	55
	14	*Avg.*	14	10	11	11	9	8	8	8	8	8	7
		SD	6	3	4	5	4	3	4	4	3	3	3
		n	57	52	51	53	49	51	53	50	55	57	75
All diets		*Avg.*	12	10	10	11	10	9	9	9	9	9	8
		SD	5	3	3	4	4	4	4	4	3	3	3
		n	791	782	660	861	771	734	757	731	766	766	1004

S2. HAEMATOLOGY AND CLINICAL BIOCHEMISTRY

Analytical methodology

Multi-parametric haematological analysis (ADVIA 120 Haematology System, Siemens Healthcare GmbH; $N = 7 - 9$ per diet) included red blood cell count (RBC), haemoglobin concentration (Hgb), haematocrit (Hct), mean corpuscular volume (MCV), mean corpuscular haemoglobin (MCH), red cell volume and haemoglobin concentration distribution widths (RDW and HDW), and white blood cell count (WBC), neutrophils, lymphocytes, monocytes, eosinophils, basophils and large unstained cell counts. Clinical biochemistry analyses ($N = 5 - 8$ per diet) for C-reactive protein, gamma-glutamyl transferase (GGT), creatine kinase (CK), aspartate aminotransferase (AST), lactate dehydrogenase (LDH), bilirubin, and iron (Fe) were conducted using an ABX Pentra 400 benchtop analyser (HORIBA Medical, France). The copper (Cu) concentration was determined by AAS (λ=324.8 nm; AA300, Perkin Elmer, USA).

Blood parameters results

Dietary study

At day 0, normal levels for red and white blood cells and related parameters, enzymes, proteins and trace elements were observed in pigs from all diet groups (Table S5). To identify potential dietary and pen size effects, statistical analysis (one-way ANOVA, Tukey pairwise) were carried between diet groups and between pen pairs with a Bonferroni corrected significance level of $\alpha = 0.05$ (i.e., critical value of $p < 0.0018$). The test results for all the measured blood parameters showed no significant differences between diet groups at any given time point, except for Hgb and Hct levels between NaSe-0.40 and Control-2 pigs at day 0, which were still significantly different for Hgb at day 16. This initial difference could be related to small differences in body weight for the animals at the start and during the first 2 weeks (Tables S1-S2). When comparing averages for pigs hosted in the 7- and 9-pig pens within each diet group, no significant differences were identified for any of the blood parameters.

During the trial, the RBC count and related parameters (Hgb, Hct, MCV, MCH) increased significantly, with 10-25% higher values after 77 days; whereas WBC, neutrophils, and LUC levels decreased by 18-44% significantly already by day 49-56. In contrast, eosinophils levels rapidly increased by two-fold during the first 16 days. Lymphocytes, monocytes and basophils levels showed high variability over time and, overall, remained constant. Among the investigated enzymes and specific proteins, only the GGT and AST levels significantly changed, with 20% increase and 27% decrease, respectively. Bilirubin concentration decreased by ca. 60% during the first 16 days and did not reach initial levels, whereas the concentration

of Fe and Cu did not show significant changes by the end of the trial. Despite of these time changes, the blood parameters were not significantly different between diet groups.

LPS challenge study

The levels of most RBC and WBC parameters and trace elements clearly changed over time after the LPS injection (Table S5). Within 3 h of injecting LPS, the WBC and all related parameters decreased by 61-90% from initial values, remaining low for another 2 to 8 h before a noticeable increase was observed (initial values were reached only after 48 h). A decrease of RBC, Hgb and Hct was also observed but became only significant after 5 h, reaching minimum values between 8 and 24 h. Despite a slight recover between 24 and 48 h, the levels did not reach initial values within this monitoring period. Other RBC related parameters showed no clear changes over time. Moreover, during the first 5 h, the Fe and Cu concentrations decreased by 60 and 10%, respectively, and bilirubin levels increased by > 100%, yet returning to initial levels by 24 h; whereas CRP and AST concentrations gradually increased without recovering to initial levels. Other enzymes and specific proteins showed no significant changes over time, except for the LDH levels of Control-1 diet group, which increased between 5-12 h after the LPS injection as well as being significantly different to the other diet groups. None of the other parameters (i.e., of red and white blood cells and related parameters, enzymes, specific proteins, metabolites and trace elements) showed significant differences between diet group averages or between averages of pigs hosted in the 7- and 9-pig pens within a diet group.

Table S5. Average levels (1 standard deviation (*SD*)) of **red and white blood cells and related parameters, enzymes, specific proteins, metabolites and trace elements** measured in pigs of all diet groups at the start of the trial (day 0), after 16, 49-56, and 77 days into the trial ($N = 72$; $n = 24 – 71$ measurements per variable), and 3, 5, 8, 12, 24 and 48 h after receiving an injection of LPS (2 µg/kg BW) at 49-56 days into the trial ($N = 67 – 69$; $n = 45 – 69$ measurements per variable).

		**Average (SD)**									
		**Dietary study** [c]				**LPS study** [c]					
Variable [a]	**Ref. interval** [b]	**Day 0**	**Day 16**	**Day 49-56**	**Day 77**	**Hour 3**	**Hour 5**	**Hour 8**	**Hour 12**	**Hour 24**	**Hour 48**
RBC, x10^{12}/L	6.2-8.6	7.0 (0.4)	7.1 (0.4)	7.4 (0.4)[#]	7.7 (0.4)[^]	7.6 (0.4)[#]	7.2 (0.5)[#]	6.8 (0.4)[^]	6.9 (0.4)[^]	6.9 (0.4)[^]	7.0 (0.4)[^]
Hgb, g/L	103-137	113 (8)	117 (7)[#]	133 (7)[^]	141 (7)[^]	136 (8)[#]	130 (9)[#]	125 (7)[^]	123 (8)[^]	123 (8)[^]	125 (6)[^]
Hct, %	31-44	37 (2)	39 (2)[#]	42 (2)[^]	44 (2)[^]	43 (2)[#]	40 (3)	38 (2)[^]	38 (2)[^]	39 (3)[^]	39 (2)[^]
MCV, fL	47-60	54 (2)	54 (4)[#]	56 (2)[^]	57 (2)[^]	56 (2)	56 (2)	56 (2)[#]	55 (2)[^]	56 (2)	56 (4)
MCH, pg	14-20	16 (1)	16 (1)	18 (1)[^]	18 (1)[^]	18 (1)	18 (1)	18 (1)[#]	18 (1)[^]	18 (1)	18 (1)
RDW, %	15-25	20 (2)	18 (1)[^]	16 (1)[^]	15 (1)[^]	16 (1)[#]	16 (1)[#]	16 (1)[^]	16 (1)[^]	16 (1)[#]	16 (1)
HDW, g/L	14-23	19 (1)	18 (1)[#]	17 (1)[^]	17 (1)[^]	17 (1)	17 (1)	17 (1)	17 (1)[^]	17 (1)	18 (1)[^]
WBC, x10^{9}/L	17.4-22.8	21 (5)	20 (4)	17 (3)[^]	18 (3)[^]	5 (2)[^]	7 (3)[^]	12 (4)[^]	14 (3)[^]	16 (3)[#]	18 (3)
Neut, x10^{9}/L	5.4-24.5	9.0 (3.8)	7.5 (2.5)[#]	5.1 (1.4)[^]	5.7 (1.5)[^]	1.8 (1.3)[^]	4.3 (2.3)[#]	8.0 (3.0)[^]	6.2 (2.1)[^]	5.1 (1.6)	4.5 (1.3)[#]
Lymf, x10^{9}/L	6.1-22.4	10 (3)	11 (3)	10 (2)	11 (2)	3 (1)[^]	2 (1)[^]	4 (2)[^]	7 (2)[^]	9 (2)[^]	11 (2)[#]
Mono, x10^{9}/L	0.4-3.7	0.53 (0.26)	0.62 (0.21)[#]	0.59 (0.19)	0.67 (0.16)[#]	0.06 (0.03)[^]	0.06 (0.04)[^]	0.09 (0.04)[^]	0.15 (0.08)[^]	0.37 (0.16)[^]	0.92 (0.27)[^]
Eos, x10^{9}/L	0.1-2.5	0.29 (0.11)	0.62 (0.30)[^]	0.61 (0.21)[^]	0.69 (0.25)[^]	0.11 (0.08)[^]	0.13 (0.08)[^]	0.22 (0.11)[^]	0.28 (0.10)[^]	0.70 (0.32)[#]	0.67 (0.23)
Baso, x10^{9}/L	0.1-0.6	0.13 (0.07)	0.12 (0.04)	0.13 (0.06)	0.16 (0.06)[#]	0.04 (0.02)[^]	0.04 (0.02)[^]	0.06 (0.03)[^]	0.07 (0.03)[^]	0.11 (0.05)	0.16 (0.05)[#]
LUC, x10^{9}/L	0.1-1.6	0.16 (0.10)	0.10 (0.06)[#]	0.10 (0.05)[#]	0.11 (0.06)[#]	0.03 (0.02)[^]	0.03 (0.02)[^]	0.07 (0.04)[#]	0.10 (0.05)[^]	0.15 (0.08)[#]	0.11 (0.06)
CRP, mg/L	-	23 (15)	24 (14)	22 (11)	20 (9)	23 (5) [d]	28 (16)	29 (4) [d]	29 (13)[#]	28 (10)[#]	36 (20) [d]
GGT, U/L	14-95	40 (17)	43 (16)	45 (16)[#]	48 (20)[#]	62 (10) [d]	43 (15)[^]	52 (11) [d]	41 (17)[#]	45 (17)	59 (12) [d]
CK, U/L	359-28155	1972 (877)	2322 (1575)	2166 (1357)	1733 (1185)	4124 (2565) [d]	2642 (2117)	5018 (2121) [d]	3705 (3132)	2338 (1552)	3571 (2942) [d]
AST, U/L	25-142	57 (16)	46 (9)[^]	41 (11)[^]	42 (16)[^]	53 (17) [d]	55 (12)[^]	70 (20) [d]	71 (23)[^]	57 (19)[#]	61 (36) [d]
LDH, U/L	521-2236	564 (185)	532 (78)	508 (95)	503 (109)	608 (123) [d]	479 (99)	607 (117) [d]	568 (171)	529 (177)	630 (175) [d]
Bili, µmol/L	0-2	1.4 (0.7)	0.55 (0.46)[#]	0.74 (0.31)[#]	0.75 (0.39)[#]	3.2 (2.8) [d]	2.5 (1.6)[^]	2.7 (1.6) [d]	1.4 (0.6)[^]	1.2 (0.5)[^]	0.94 (0.34) [d]
Fe, µmol/L	8-57	32 (12)	22 (10)[#]	28 (8)	29 (5)	20 (6) [d]	10 (4)[^]	7 (3) [d]	11 (5)[^]	26 (7)	25 (7) [d]
Cu, µmol/L	21-44	33 (4)	n.m.	29 (3)[^]	31 (4)	27 (2) [d]	26 (3)[^]	27 (2) [d]	28 (3)[#]	28 (3)[#]	30 (3) [d]

[a] Red blood cell count (RBC), haemoglobin concentration (Hgb), haematocrit (Hct), mean corpuscular volume (MCV), mean corpuscular haemoglobin (MCH), red cell volume and haemoglobin concentration distribution widths (RDW and HDW), white blood cell count (WBC), neutrophils (Neut), lymphocytes (Lymf), monocytes (Mono), eosinophils (Eos), basophils (Baso), large unstained cell count (LUC), C-reactive protein (CRP), gamma-glutamyl transferase (GGT), creatine kinase (CK), aspartate aminotransferase (AST), lactate dehydrogenase (LDH), bilirubin (Bili), iron (Fe), copper (Cu).

[b] Typical reference intervals for Norwegian crossbreed grower pigs [2].

[c] Significance test with respect to initial values (paired t-test, two-tailed distribution; $^{\#}p < 0.05$, $^{\wedge}p < 0.0018$) for the differences between: Day 0 – Day 16, Day 0 – Day 49-56, Day 0 – Day 77, and Hour 0 (i.e., Day 49-56) – Hour 3, Hour 0 – Hour 5, Hour 0 – Hour 8, Hour 0 – Hour 12, Hour 0 – Hour 24, Hour 0 – Hour 48.

[d] Only measured in plasma of pigs from Control-1 diet group ($n = 8$), not possible to carry out significance test with respect to initial values or between diet groups.

S3. CLINICAL EXAMINATIONS

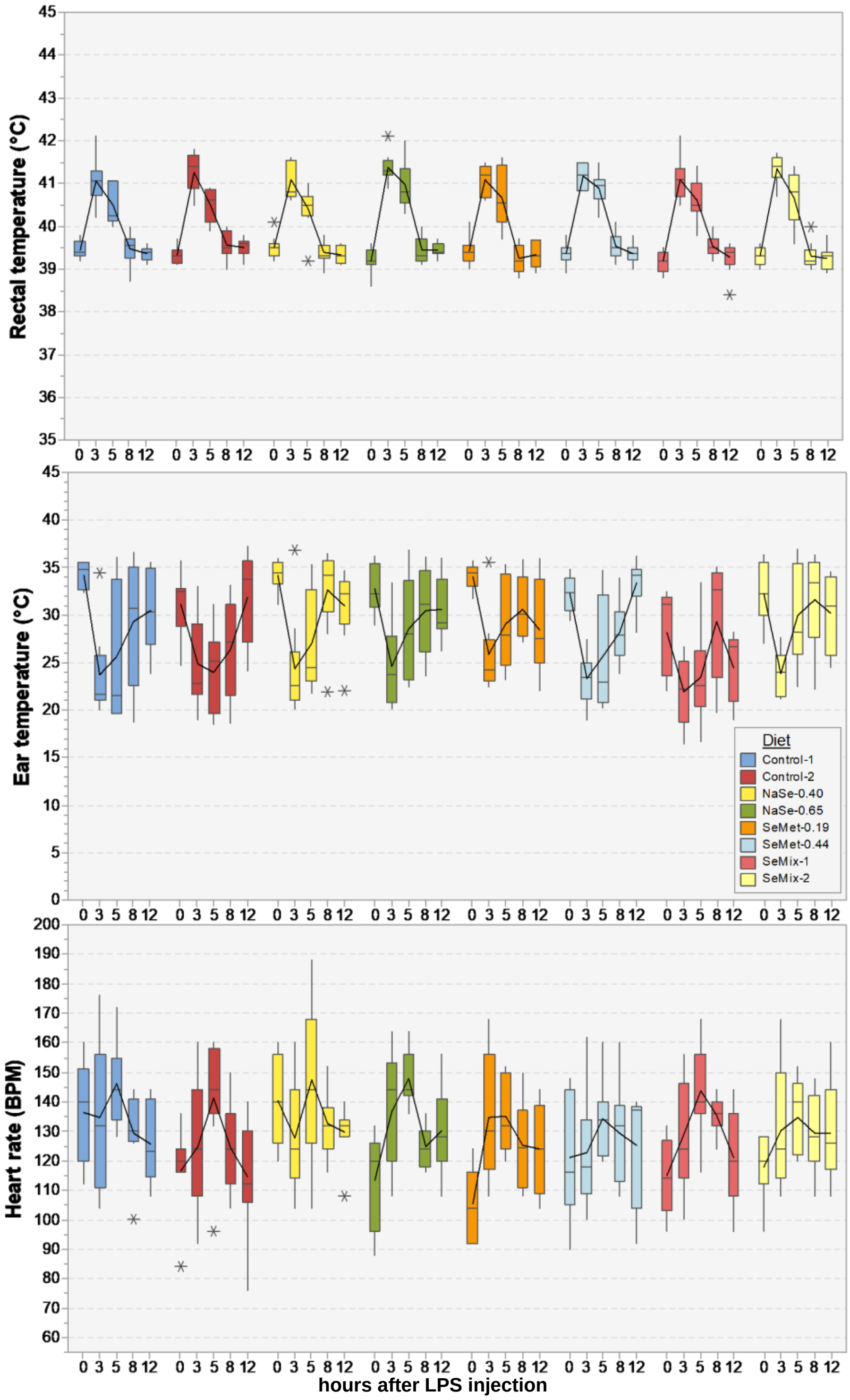

Figure S3. Boxplot of rectal temperature (*top*), average temperature of left and right ears (*middle*) and heart rate (*bottom*) of pigs ($N = 9$ per diet) fed with non-Se-supplemented (Control) and NaSe and/or SeMet enriched diets (Table 1) before and 3, 5, 8 and 12 h after receiving an injection of LPS (2 µg/kg BW) at 49-56 days into the trial (x-axes, non-linear scale). Box plots are connected by the mean at each time point (solid black line). No significant differences were observed between averages of diet groups at any given time point (one-way ANOVA, Tukey pairwise, 99.82% confidence level).

S4. TOTAL SELENIUM, VITAMIN E, AND SELENIUM SPECIATION

Analytical methodology

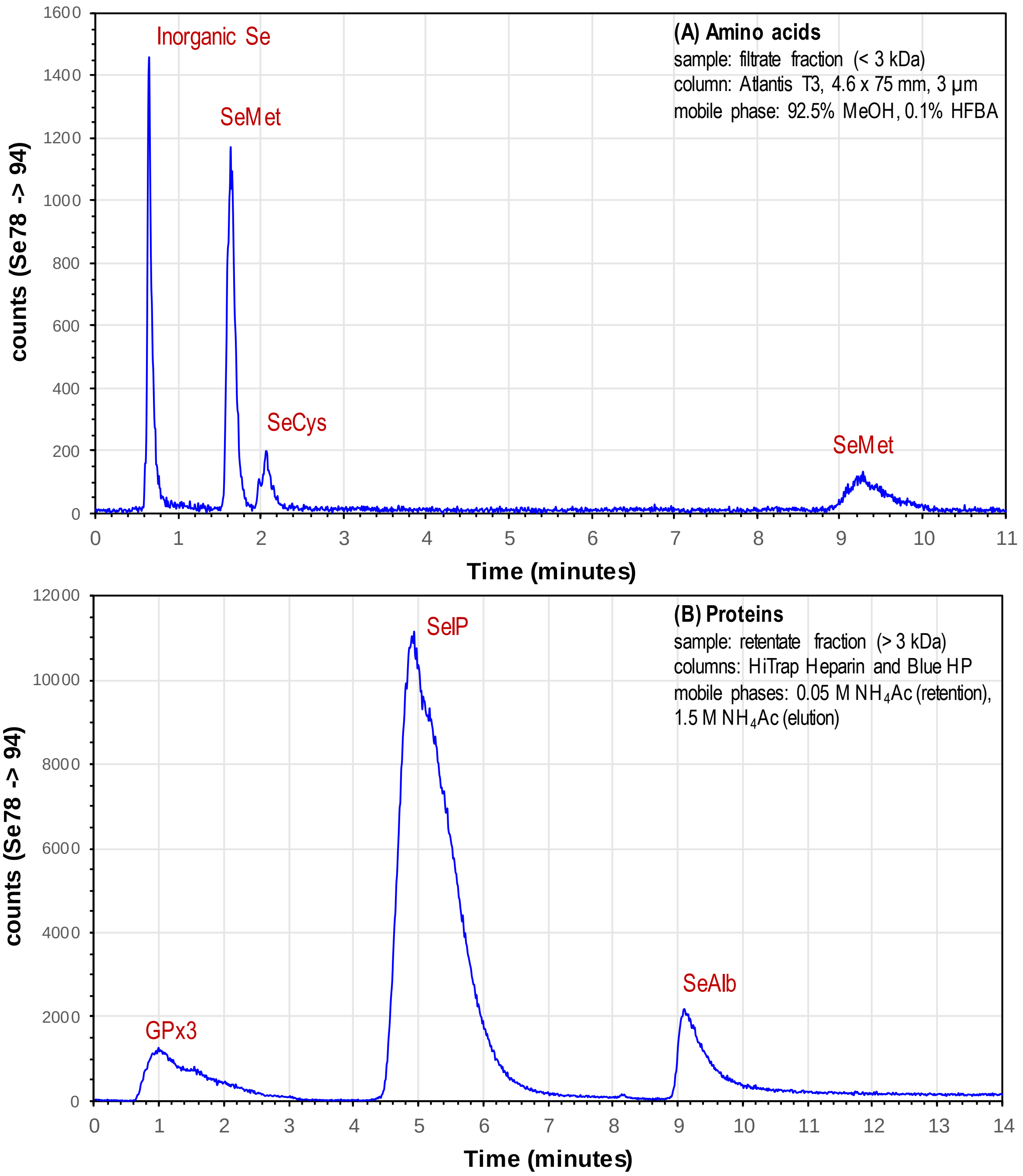

Figure S4. Representative HPLC-ICP-MS chromatograms for the **(A)** < 3 kDa (inorganic Se and seleno amino acids) and **(B)** > 3 kDa (Se-containing proteins) fractions of pigs' plasma. Retention time is in minutes and signal intensity is in counts for the monitored mass pair $^{78}Se^{+} \rightarrow {}^{78}Se^{16}O^{+}$. Integrated peak areas were used for the quantification of Se associated to the selenospecies. Analytical methodology is explained in detail in the methods section.

Results

Plasma concentrations during the dietary study

Table S6. Average concentration (1 standard deviation (SD), *n* = number of measurements) of **total Se and vitamin E** determined **in plasma** of pigs fed non-Se-supplemented (Control) and NaSe and/or SeMet enriched diets (Table 1) at the start (day 0) and 16, 49-56 and 77 days into the trial ($N = 5 – 9$ pigs per diet). Average concentrations of Se (in µg/L of plasma) or vitamin E (in µg/mL of plasma) within a given time point that do not share a grouping letter are significantly different (one-way ANOVA, Tukey pairwise, 99.82% confidence level). Data boxplots are presented in Figure 1.

	**Day 0**				**Day 16**				**Day 49-56**				**Day 77**			
Diet group	***n***	**Avg.**	**SD**	**grouping**	***n***	**Avg.**	**SD**	**grouping**	***n***	**Avg.**	**SD**	**grouping**	***n***	**Avg.**	**SD**	**grouping**
Total Se, µg/L																
Control-1	9	140	15	A	9	105	5	DE	5	110	19	BC	8	150	25	A
Control-2	9	139	15	A	9	91	14	E	5	101	19	C	9	159	26	A
NaSe-0.40	9	129	8	A	9	143	17	BC	5	149	17	ABC	9	160	30	A
NaSe-0.65	9	131	15	A	9	156	12	AB	5	158	15	AB	9	169	26	A
SeMet-0.19	9	131	10	A	9	125	14	CD	5	142	24	ABC	9	195	24	A
SeMet-0.44	9	136	22	A	9	186	25	A	5	189	9	A	9	198	20	A
SeMix-1	9	134	13	A	8	175	4	A	5	183	15	A	8	202	24	A
SeMix-2	9	138	12	A	9	173	18	AB	5	173	26	A	9	192	31	A
Vitamin E, µg/mL																
Control-1	7	2.75	0.52	A	7	1.40	0.25	B					7	1.56	0.18	C
Control-2	7	2.28	0.45	A	7	1.16	0.36	B					7	2.81	0.31	B
NaSe-0.40	7	2.94	0.81	A	7	1.63	0.49	AB					7	2.57	0.59	BC
NaSe-0.65	7	3.00	0.38	A	7	1.86	0.26	AB					7	3.49	0.49	AB
SeMet-0.19	7	2.45	0.42	A	7	1.64	0.43	AB					7	3.19	0.64	B
SeMet-0.44	7	2.66	0.43	A	7	1.83	0.71	AB					7	3.64	0.41	AB
SeMix-1	7	3.03	0.62	A	7	1.57	0.50	B					7	2.96	0.64	B
SeMix-2	7	3.07	0.84	A	7	2.82	0.93	A					7	4.46	0.66	A

Table S7. Average concentration (1 standard deviation (SD), *n* = number of measurements) of **selenospecies (inorganic, amino acids, proteins)** determined **in plasma** of pigs fed non-Se-supplemented (Control) and NaSe and/or SeMet enriched diets (Table 1) at the start (day 0) and 16, 49-56 and 77 days into the trial (*N* = 5 – 9 pigs per diet). Average concentrations (in µg Se/L of plasma) within a given time point that do not share a grouping letter are significantly different (one-way ANOVA, Tukey pairwise, 99.82% confidence level). Data boxplots are presented in Figure 2.

	**Day 0**				**Day 16**				**Day 49-56**				**Day 77**			
Diet group	***n***	**Avg.**	**SD**	**grouping**	***n***	**Avg.**	**SD**	**grouping**	***n***	**Avg.**	**SD**	**grouping**	***n***	**Avg.**	**SD**	**grouping**
Inorganic Se, µg Se/L																
Control-1	9	0.75	0.13	A	9	0.76	0.24	AB	5	0.64	0.04	BC	9	1.00	0.11	CD
Control-2	9	0.75	0.13	A	9	0.64	0.18	B	5	0.58	0.02	C	9	0.89	0.10	D
NaSe-0.40	8	0.72	0.09	A	9	0.80	0.28	AB	5	0.78	0.09	AB	9	1.32	0.17	ABC
NaSe-0.65	9	0.68	0.11	A	9	1.00	0.20	A	5	0.81	0.04	A	7	1.56	0.24	A
SeMet-0.19	9	0.70	0.10	A	9	0.60	0.08	B	5	0.67	0.05	ABC	9	1.12	0.09	BCD
SeMet-0.44	8	0.66	0.12	A	9	0.66	0.05	AB	5	0.67	0.04	ABC	8	1.07	0.11	BCD
SeMix-1	9	0.76	0.12	A	9	0.81	0.09	AB	5	0.73	0.07	ABC	7	1.38	0.17	ABC
SeMix-2	9	0.89	0.16	A	9	0.83	0.20	AB	5	0.67	0.04	ABC	9	1.38	0.33	AB
SeCys, µg Se/L																
Control-1	9	0.46	0.12	A	9	0.48	0.13	A	5	0.56	0.02	AB	9	0.52	0.06	B
Control-2	9	0.52	0.15	A	9	0.46	0.11	A	5	0.52	0.02	B	9	0.51	0.05	B
NaSe-0.40	9	0.51	0.14	A	9	0.53	0.18	A	5	0.64	0.03	AB	9	0.62	0.05	AB
NaSe-0.65	9	0.46	0.11	A	9	0.66	0.19	A	5	0.71	0.09	A	8	0.72	0.13	A
SeMet-0.19	9	0.45	0.13	A	9	0.48	0.13	A	5	0.57	0.04	AB	9	0.58	0.08	AB
SeMet-0.44	7	0.45	0.15	A	9	0.54	0.17	A	5	0.63	0.02	AB	9	0.66	0.16	AB
SeMix-1	9	0.52	0.14	A	9	0.57	0.17	A	5	0.60	0.05	AB	7	0.63	0.05	AB
SeMix-2	9	0.54	0.15	A	9	0.53	0.19	A	5	0.65	0.04	AB	9	0.62	0.07	AB
SeMet, µg Se/L																
Control-1	9	0.58	0.16	A	8	0.24	0.01	C	5	0.25	0.01	C	9	0.37	0.15	D
Control-2	9	0.50	0.15	A	8	0.23	0.01	C	5	0.23	0.01	C	9	0.34	0.15	D
NaSe-0.40	9	0.64	0.19	A	9	0.60	0.19	BC	5	0.86	0.27	BC	9	0.99	0.31	BC
NaSe-0.65	9	0.53	0.14	A	9	1.21	0.50	A	5	2.09	0.40	A	8	2.07	0.56	A
SeMet-0.19	9	0.47	0.10	A	9	0.37	0.06	C	5	0.55	0.18	C	8	0.74	0.13	CD
SeMet-0.44	8	0.53	0.18	A	9	0.98	0.28	AB	5	1.49	0.24	AB	9	1.56	0.33	AB
SeMix-1	9	0.61	0.14	A	9	0.93	0.21	AB	5	1.57	0.27	AB	7	1.80	0.27	A
SeMix-2	9	0.616	0.06	A	9	1.02	0.34	AB	5	1.34	0.38	AB	9	1.61	0.16	A

Table S7. ***(continued)***

Diet group	Day 0 *n*	Avg.	SD	grouping	Day 16 *n*	Avg.	SD	grouping	Day 49-56 *n*	Avg.	SD	grouping	Day 77 *n*	Avg.	SD	grouping
GPx3, μg Se/L																
Control-1	9	10.8	4.8	A	9	9.6	2.2	B	5	9.5	5.1	AB	9	12.4	5.2	B
Control-2	9	10.4	2.1	A	9	8.3	2.5	B	5	7.3	5.1	B	9	12.3	7.0	B
NaSe-0.40	9	10.5	3.5	A	9	12.4	6.5	B	4	11.2	1.1	AB	9	13.9	4.2	B
NaSe-0.65	9	10.7	2.6	A	9	12.6	7.7	B	5	15.3	5.1	AB	8	15.3	4.6	B
SeMet-0.19	9	12.6	4.0	A	9	14.0	3.9	B	5	14.3	5.0	AB	9	15.6	4.7	B
SeMet-0.44	9	10.0	2.3	A	9	24.1	3.1	A	5	21.7	3.5	A	9	25.4	1.7	A
SeMix-1	9	11.2	3.7	A	9	13.3	4.7	B	5	18.7	3.8	AB	7	17.5	4.8	AB
SeMix-2	9	11.4	1.7	A	9	15.0	5.4	AB	5	17.1	4.9	AB	9	18.4	4.2	AB
SelP, μg Se/L																
Control-1	9	115	19	A	9	71	12	CD	5	67	19	A	9	103	21	AB
Control-2	9	117	13	A	9	57	7	D	5	67	12	A	9	94	28	B
NaSe-0.40	9	99	11	AB	9	97	15	ABC	5	89	28	A	9	113	21	AB
NaSe-0.65	9	92	15	AB	9	108	16	AB	5	99	9	A	8	125	18	AB
SeMet-0.19	9	104	8	AB	9	73	13	BCD	5	96	21	A	9	121	17	AB
SeMet-0.44	9	85	20	B	9	99	14	ABC	4	107	1	A	9	135	16	A
SeMix-1	9	100	14	AB	9	107	36	ABC	5	107	10	A	7	141	15	A
SeMix-2	9	100	11	AB	9	115	19	A	5	88	27	A	9	127	14	AB
SeAlb, μg Se/L																
Control-1	9	9.6	1.3	A	9	7.0	1.3	AB	5	5.7	1.5	CD	9	6.2	1.4	D
Control-2	9	10.4	3.6	A	9	6.4	2.0	B	5	3.5	0.5	D	9	6.0	2.1	D
NaSe-0.40	9	8.7	1.6	A	9	9.1	2.1	AB	5	12.8	5.0	AB	9	11.8	2.0	BC
NaSe-0.65	9	10.1	1.4	A	9	10.5	3.3	AB	5	14.2	2.8	A	8	16.1	2.2	A
SeMet-0.19	9	8.9	1.9	A	9	7.7	2.1	AB	5	6.7	1.0	BCD	9	9.9	1.1	C
SeMet-0.44	9	7.7	1.5	A	9	11.4	2.4	A	5	12.1	0.6	ABC	9	15.0	1.6	AB
SeMix-1	9	10.1	1.0	A	9	10.5	2.2	AB	5	12.0	1.4	ABC	7	15.6	2.3	A
SeMix-2	9	9.8	0.8	A	9	10.9	2.7	AB	5	11.5	2.0	ABC	9	14.1	0.9	AB

Plasma concentrations during the LPS challenge study

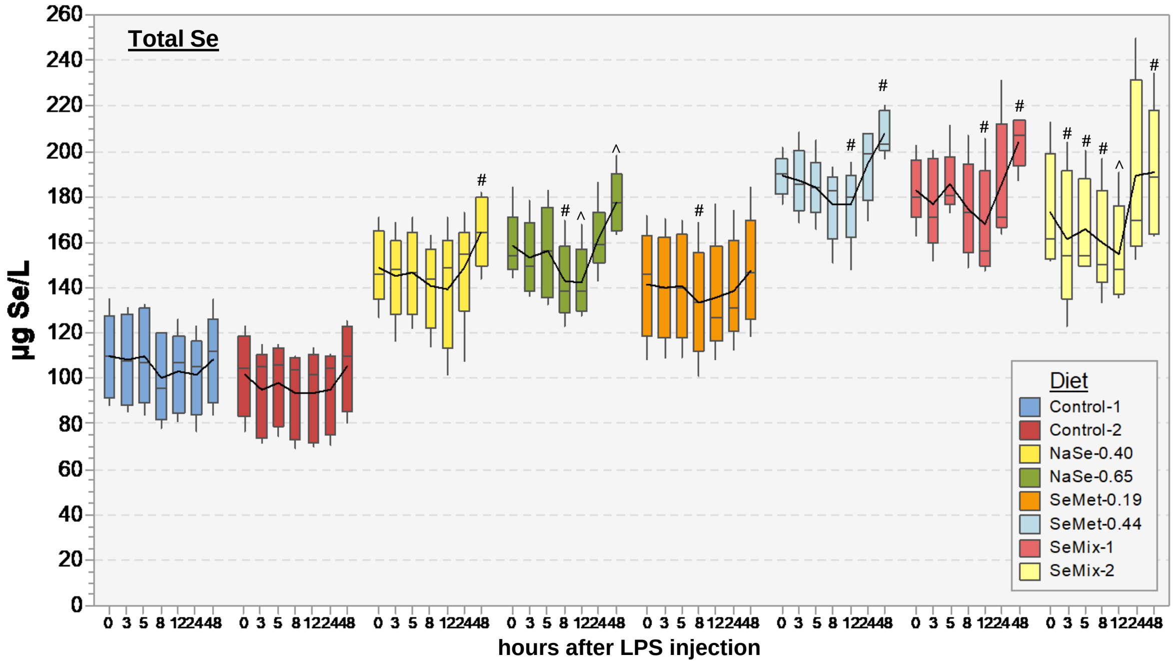

Figure S5. Concentration of **total Se** (in µg/L; y-axis) **in plasma** of pigs (N = 5 per diet) fed non-Se-supplemented (Control) and NaSe and/or SeMet enriched diets (Table 1) before and 3, 5, 8, 12, 24 and 48 h after receiving a LPS injection (2 µg/kg BW) at 49-56 days into the trial (x-axes, non-linear scale). Box plots are connected by the mean at each time point (solid black line), whose significant differences with respect to time 0 h are indicated with # for $p < 0.05$ and ^ for $p < 0.00178$ (paired t-test, two-tailed distribution). Average concentrations, associated standard deviations, and significance test grouping between diet groups within a given time point are presented in Table S8.

Table S8. Average concentration (1 standard deviation (SD), *n* = number of measurements) of **total Se and selenospecies (inorganic, amino acids, proteins)** determined **in plasma** of pigs fed non-Se-supplemented (Control) and NaSe and/or SeMet enriched diets (Table 1) 3, 5, 8, 12, 24 and 48 h after receiving a 2 µg LPS/kg BW injection at 49-56 days into the trial (*N* = 5 per diet). Data for time 0 h can be found as Day 49-56 in Table S6 and S8. Average concentrations within a given time point that do not share a grouping letter are significantly different (one-way ANOVA, Tukey pairwise, 99.82% confidence level). Time series and significance between time points are represented in Figures S5 and Figures 4 and 5.

	**3 h**				**5 h**				**8 h**				**12 h**				**24 h**				**48 h**			
Diet group	***n***	**Avg.**	**SD**	**group**	***n***	**Avg.**	**SD**	**group**	***n***	**Avg.**	**SD**	**group**	***n***	**Avg.**	**SD**	**group**	***n***	**Avg.**	**SD**	**group**	***n***	**Avg.**	**SD**	**group**
Total Se, µg/L																								
Control-1	5	108	20	BC	5	109	21	BC	5	100	19	B	5	103	18	B	5	101	18	B	5	109	20	C
Control-2	5	95	20	C	5	98	18	C	5	93	19	B	5	93	20	B	5	95	18	B	5	105	19	C
NaSe-0.40	5	145	19	ABC	5	146	19	ABC	5	141	19	AB	5	139	27	AB	5	149	24	AB	5	165	16	AB
NaSe-0.65	5	153	17	ABC	5	156	21	ABC	5	143	17	AB	5	142	16	AB	5	161	16	AB	5	178	14	AB
SeMet-0.19	5	140	24	ABC	5	140	24	ABC	5	134	25	AB	5	135	26	AB	5	139	23	AB	5	148	25	BC
SeMet-0.44	5	187	15	A	5	184	14	A	5	176	16	A	5	177	18	A	5	194	16	A	5	208	10	A
SeMix-1	5	177	20	A	5	179	25	A	5	174	22	A	5	168	24	A	5	186	28	A	5	204	11	A
SeMix-2	5	161	31	AB	4	165	22	AB	5	160	24	A	5	165	23	AB	5	190	41	A	5	191	30	AB
Inorganic Se, µg Se/L																								
Control-1	5	0.58	0.06	CD	5	0.85	0.14	CD	5	0.99	0.11	BC	5	0.79	0.08	B	5	0.57	0.01	AB	5	0.56	0.02	B
Control-2	5	0.56	0.03	D	5	0.72	0.08	D	5	0.78	0.10	C	5	0.74	0.09	B	5	0.55	0.03	B	5	0.55	0.02	B
NaSe-0.40	5	0.74	0.06	AB	5	1.12	0.10	ABC	5	1.14	0.13	AB	5	1.10	0.13	AB	5	0.68	0.07	AB	5	0.72	0.02	AB
NaSe-0.65	5	0.83	0.07	A	5	1.33	0.18	A	5	1.42	0.16	A	5	1.38	0.28	A	5	0.82	0.13	AB	5	0.85	0.12	A
SeMet-0.19	5	0.63	0.04	BCD	5	0.92	0.07	BCD	5	1.06	0.12	BC	5	1.05	0.09	AB	5	0.59	0.04	AB	5	0.53	0.11	B
SeMet-0.44	5	0.66	0.03	BCD	5	1.03	0.13	ABCD	5	1.21	0.18	AB	5	1.13	0.15	AB	5	0.84	0.21	A	5	0.66	0.03	AB
SeMix-1	5	0.72	0.06	ABC	5	1.24	0.14	AB	5	1.28	0.12	AB	5	1.23	0.15	A	5	0.76	0.07	AB	5	0.69	0.11	AB
SeMix-2	5	0.68	0.03	BCD	5	0.99	0.11	AB	5	1.05	0.05	BC	5	1.06	0.11	A	5	0.61	0.03	AB	5	0.68	0.02	AB

Table S8. ***(continued)***

	3 h				5 h				8 h				12 h				24 h				48 h			
Diet group	***n***	**Avg.**	**SD**	**group**	***n***	**Avg.**	**SD**	**group**	***n***	**Avg.**	**SD**	**group**	***n***	**Avg.**	**SD**	**group**	***n***	**Avg.**	**SD**	**group**	***n***	**Avg.**	**SD**	**group**
SeCys, µg Se/L																								
Control-1	5	0.56	0.02	DE	5	0.55	0.03	CD	5	0.58	0.00	BC	5	0.52	0.03	BC	5	0.59	0.01	B	5	0.58	0.01	CD
Control-2	5	0.54	0.03	E	5	0.49	0.01	D	5	0.54	0.02	C	5	0.50	0.01	C	5	0.56	0.02	B	5	0.55	0.02	D
NaSe-0.40	5	0.68	0.04	BC	5	0.62	0.02	ABC	5	0.62	0.04	ABC	5	0.58	0.03	AB	5	0.66	0.05	AB	5	0.74	0.02	AB
NaSe-0.65	5	0.83	0.05	A	5	0.69	0.07	A	5	0.72	0.06	A	5	0.65	0.05	A	5	0.76	0.09	A	5	0.84	0.05	A
SeMet-0.19	5	0.59	0.04	CDE	5	0.55	0.04	CD	5	0.57	0.05	BC	5	0.54	0.02	BC	5	0.58	0.04	B	5	0.61	0.10	BCD
SeMet-0.44	5	0.66	0.04	BCD	5	0.57	0.01	BCD	5	0.66	0.03	AB	5	0.60	0.02	AB	5	0.79	0.09	A	5	0.72	0.02	ABC
SeMix-1	5	0.69	0.03	BC	5	0.66	0.04	AB	5	0.66	0.04	AB	5	0.57	0.02	BC	5	0.66	0.07	AB	5	0.73	0.06	AB
SeMix-2	5	0.70	0.04	B	5	0.61	0.01	ABC	5	0.65	0.04	AB	5	0.55	0.03	BC	5	0.68	0.03	AB	5	0.72	0.05	ABC
SeMet, µg Se/L																								
Control-1	5	0.25	0.02	DE	5	0.46	0.08	BC	5	0.54	0.12	B	5	0.59	0.02	C	5	0.26	0.02	B	5	0.28	0.01	C
Control-2	5	0.23	0.00	E	5	0.29	0.05	C	5	0.39	0.07	B	5	0.57	0.04	C	5	0.24	0.02	B	5	0.26	0.01	C
NaSe-0.40	5	0.92	0.23	CD	5	0.84	0.20	B	5	0.90	0.23	B	5	1.04	0.16	BC	5	0.68	0.42	AB	5	0.88	0.20	BC
NaSe-0.65	5	2.82	0.28	A	5	1.76	0.13	A	5	1.56	0.17	A	5	1.58	0.29	AB	5	0.87	0.50	AB	5	1.84	0.40	A
SeMet-0.19	5	0.57	0.19	DE	5	0.73	0.16	B	5	0.84	0.19	B	5	0.94	0.12	BC	5	0.43	0.08	AB	5	0.46	0.13	C
SeMet-0.44	5	1.36	0.22	BC	5	1.40	0.18	A	5	1.82	0.23	A	5	1.95	0.55	A	5	1.33	0.62	A	5	1.52	0.37	AB
SeMix-1	5	1.74	0.43	B	5	1.57	0.15	A	5	1.64	0.18	A	5	1.66	0.17	AB	5	0.89	0.20	AB	5	1.63	0.28	AB
SeMix-2	5	1.74	0.20	B	5	1.41	0.18	A	5	1.59	0.26	A	5	1.46	0.30	AB	5	0.72	0.16	AB	5	1.25	0.42	AB
GPx3, µg Se/L																								
Control-1	5	8.87	5.22	BC	5	7.84	5.51	C	5	12.3	3.9	B	5	11.0	5.9	D	5	28.0	8.5	A	5	23.2	6.9	D
Control-2	5	9.62	4.70	BC	5	8.36	3.30	C	5	12.5	3.3	B	5	10.2	2.5	D	5	25.3	6.3	A	5	26.1	6.4	CD
NaSe-0.40	5	7.44	4.19	CD	5	7.97	3.17	C	4	13.8	3.0	B	5	12.0	3.5	D	5	26.3	9.1	A	5	32.8	10.8	BCD
NaSe-0.65	5	8.75	3.00	BC	5	11.5	5.2	BC	5	16.5	2.7	B	5	13.5	0.9	CD	5	31.5	4.8	A	5	41.4	4.9	BC
SeMet-0.19	5	10.7	4.6	ABC	5	15.3	3.4	ABC	5	16.6	6.3	B	5	17.1	2.9	BCD	5	30.3	5.7	A	5	45.8	10.8	AB
SeMet-0.44	5	18.8	3.8	A	5	23.7	4.5	A	5	29.5	3.9	A	5	24.6	4.4	A	5	29.7	15.8	A	5	59.6	6.2	A
SeMix-1	5	17.2	3.2	AB	5	18.8	6.1	AB	5	25.7	4.2	A	5	20.0	3.1	AB	5	37.9	3.9	A	5	38.2	4.0	BC
SeMix-2	5	15.6	5.4	AB	5	18.0	3.6	AB	5	27.9	3.4	A	5	21.8	2.8	AB	5	34.7	8.6	A	5	41.3	7.9	BC

Table S8. ***(continued)***

	**3 h**				**5 h**				**8 h**				**12 h**				**24 h**				**48 h**			
Diet group	***n***	**Avg.**	**SD**	**group**	***n***	**Avg.**	**SD**	**group**	***n***	**Avg.**	**SD**	**group**	***n***	**Avg.**	**SD**	**group**	***n***	**Avg.**	**SD**	**group**	***n***	**Avg.**	**SD**	**group**
SelP, µg Se/L																								
Control-1	5	82	14	A	5	58	16	B	5	54	9	A	5	54	22	A	5	79	22	A	5	73	15	C
Control-2	5	72	22	A	5	59	14	B	5	52	9	A	5	60	12	A	5	70	13	A	5	62	21	C
NaSe-0.40	5	100	20	A	5	83	12	AB	4	76	10	A	5	89	22	A	5	109	20	A	5	98	18	BC
NaSe-0.65	5	114	30	A	5	90	19	AB	5	74	11	A	5	84	15	A	5	130	14	A	5	128	16	AB
SeMet-0.19	5	108	23	A	5	89	19	AB	5	73	22	A	5	81	15	A	5	80	14	A	5	126	22	AB
SeMet-0.44	5	112	7	A	5	102	12	A	5	87	10	A	5	97	13	A	5	136	53	A	5	160	10	A
SeMix-1	5	119	18	A	5	98	12	AB	5	89	21	A	5	100	29	A	5	110	42	A	5	103	19	BC
SeMix-2	5	103	8	A	5	91	14	AB	5	82	17	A	5	84	11	A	5	116	18	A	5	101	18	BC
SeAlb, µg Se/L																								
Control-1	5	4.2	0.5	D	5	4.5	0.9	D	5	5.3	1.3	DE	5	4.9	1.4	C	5	6.4	0.5	C	5	6.5	1.3	E
Control-2	5	4.6	0.8	D	5	4.7	1.8	D	5	4.3	0.2	E	5	4.8	0.8	C	5	5.8	0.9	C	5	8.2	2.1	DE
NaSe-0.40	5	8.7	1.7	BC	5	8.9	1.0	BCD	5	9.7	1.3	BC	5	11.3	2.8	AB	5	13.1	3.5	BC	5	14.0	1.6	C
NaSe-0.65	5	13.3	1.9	A	5	14.1	2.5	A	5	13.1	0.8	A	5	15.2	1.6	A	5	16.5	2.3	AB	5	22.2	2.7	A
SeMet-0.19	5	7.8	1.3	CD	5	7.9	1.2	CD	5	7.8	1.1	CD	5	8.6	0.8	BC	5	8.6	0.9	BC	5	12.9	1.2	CD
SeMet-0.44	5	12.2	0.7	AB	5	13.1	1.8	AB	5	13.0	0.6	A	5	15.0	1.0	A	5	22.2	5.4	A	5	20.1	1.4	AB
SeMix-1	5	11.8	1.8	AB	5	12.4	0.7	ABC	5	13.3	0.8	A	5	14.3	1.3	A	5	16.3	4.5	AB	5	14.4	1.8	C
SeMix-2	5	11.2	0.7	ABC	5	12.4	2.7	ABC	5	12.0	1.1	AB	5	12.6	1.1	AB	5	15.2	2.7	AB	5	15.7	2.6	BC

S5. TISSUES POST-MORTEM

Results

Selenium and vitamin E concentrations

Table S9. Average concentration (1 standard deviation (SD), *n* = number of measurements) of **total Se and vitamin E in *post-mortem* tissues** (mg/kg dry matter) from pigs fed non-Se-supplemented (Control) and NaSe and/or SeMet enriched diets (Table 1) at the end of the 80-day dietary study (*N* = 5 – 8 pigs per diet). Averages within a given tissue that do not share a grouping letter are significantly different (one-way ANOVA, Tukey pairwise, 99.82% confidence level). Data boxplots are presented in Figure 3.

	**Brain (frontal cortex)**				**Diaphragm**				**Heart (myocardium)**				**Kidney (cortex)**				**Liver**				***M. longissimus dorsi***				***M. semimembranosus***			
Diet group	***n***	**Avg.**	**SD**	**group**	***n***	**Avg.**	**SD**	**group**	***n***	**Avg.**	**SD**	**group**	***n***	**Avg.**	**SD**	**group**	***n***	**Avg.**	**SD**	**group**	***n***	**Avg.**	**SD**	**group**	***n***	**Avg.**	**SD**	**group**
Selenium, mg/kg of dry matter																												
Control-1	5	0.58	0.04	CD	7	0.30	0.03	D	7	0.83	0.05	DE	7	8.34	0.71	B	7	1.23	0.07	D	7	0.29	0.01	E	7	0.29	0.02	E
Control-2	5	0.53	0.07	D	6	0.31	0.04	D	7	0.73	0.07	E	7	8.64	0.42	B	7	1.07	0.13	D	7	0.26	0.01	E	7	0.27	0.01	E
NaSe-0.40	5	0.61	0.07	CD	7	0.39	0.06	D	7	0.93	0.06	D	7	8.91	0.84	B	7	1.70	0.11	C	6	0.39	0.02	DE	7	0.39	0.03	DE
NaSe-0.65	5	0.58	0.04	CD	7	0.46	0.05	CD	7	0.97	0.08	D	7	8.83	0.77	B	7	1.90	0.14	BC	7	0.43	0.04	D	7	0.47	0.06	D
SeMet-0.19	5	0.76	0.06	CD	8	0.66	0.07	C	7	1.21	0.07	C	7	9.20	0.53	AB	7	1.72	0.14	C	7	0.73	0.04	C	7	0.74	0.04	C
SeMet-0.44	5	1.39	0.14	A	7	1.70	0.20	A	7	2.33	0.08	A	7	10.6	0.7	A	7	2.36	0.12	A	7	1.96	0.13	A	7	1.89	0.11	A
SeMix-1	5	1.08	0.06	B	7	1.02	0.14	B	7	1.61	0.04	B	6	10.8	0.9	A	7	2.04	0.18	B	7	1.22	0.06	B	7	1.14	0.09	B
SeMix-2	5	1.00	0.06	B	8	1.01	0.12	B	7	1.56	0.09	B	7	9.77	0.88	AB	8	2.00	0.09	B	8	1.13	0.06	B	8	1.09	0.09	B
Vitamin E, mg/kg of dry matter																												
Control-1									5	4.55	0.65	C					5	3.78	0.45	C	5	1.35	0.09	B	5	1.59	0.24	B
Control-2									5	8.22	0.66	B					5	7.06	1.58	BC	5	2.26	0.28	AB	5	2.50	0.60	AB
NaSe-0.40									5	7.71	0.83	BC					5	7.77	0.90	ABC	5	2.19	0.23	AB	5	2.39	0.63	AB
NaSe-0.65									5	9.73	0.99	AB					5	8.86	0.94	ABC	5	2.59	0.34	AB	5	3.30	0.51	A
SeMet-0.19									6	8.46	1.82	B					6	7.65	2.51	BC	6	2.35	0.62	AB	6	2.57	0.67	AB
SeMet-0.44									5	9.57	1.11	AB					5	8.40	1.50	ABC	5	2.61	0.50	A	5	3.03	0.55	AB
SeMix-1									5	10.0	1.0	AB					5	9.59	1.55	AB	5	2.89	0.36	A	5	3.18	0.34	AB
SeMix-2									6	11.8	1.4	A					6	12.9	3.41	A	6	3.21	0.64	A	6	3.70	0.79	A

Histopathology lesion scoring

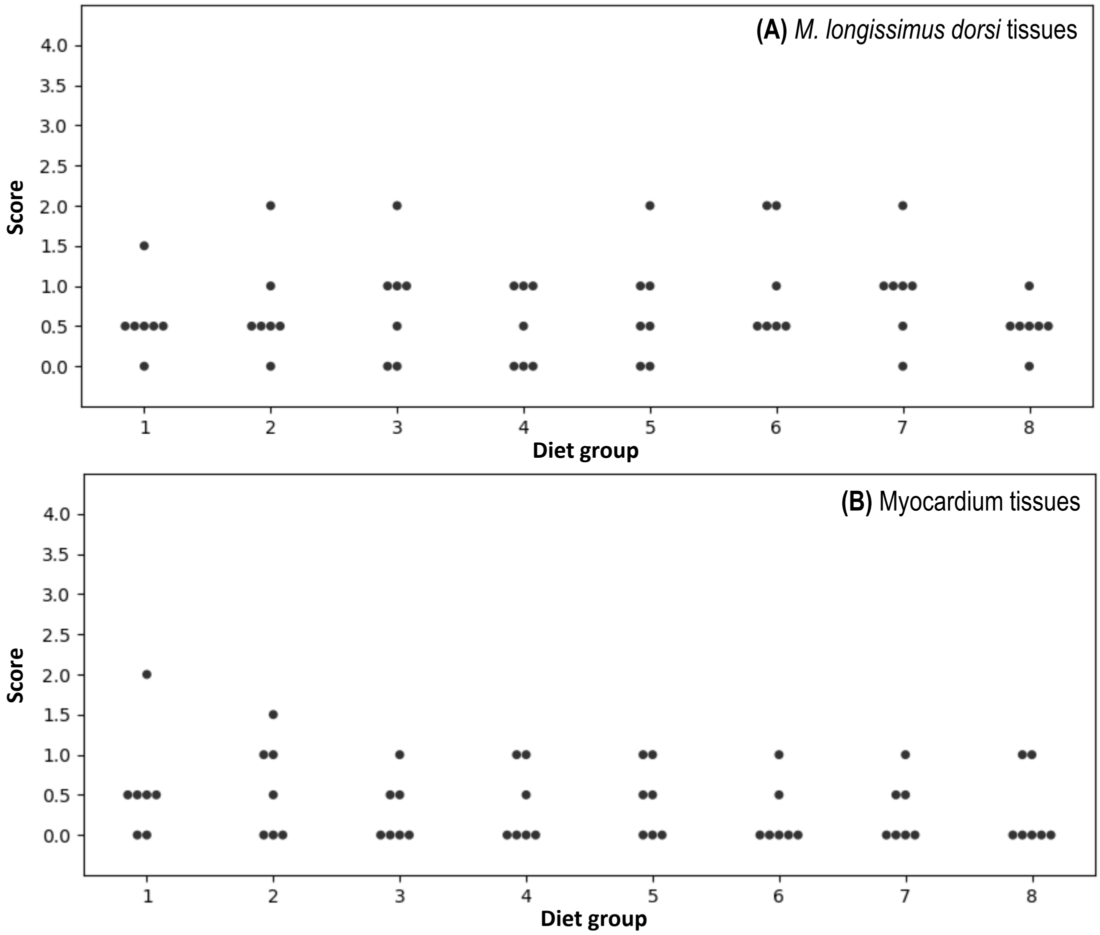

Figure S6. Scoring of histopathology changes for *M. longissimus dorsi* (**A**) and myocardium (**B**) tissues of pigs (*N* = 7 per diet) fed non-Se-supplemented (Control) and NaSe and/or SeMet enriched diets (Table 1) for 80 days. Scoring was carried out without prior knowledge of the diet group number correspondence, which was: 1 – Control-1, 2 – Control-2, 3 – SeMix-2, 4 – SeMix-1, 5 – NaSe-0.40, 6 – NaSe-0.65, 7 – SeMet-0.19, and 8 – SeMet-0.44. Lesions scoring scale: 0 – none or minor changes, 1 – mild changes, 2 – moderate changes, 3 – major changes, and 4 – severe changes.

REFERENCES

1. Collins CL, Pluske JR, Morrison RS, McDonald TN, Smits RJ, Henman DJ, et al. Post-weaning and whole-of-life performance of pigs is determined by live weight at weaning and the complexity of the diet fed after weaning. Animal Nutrition. 2017;3(4):372-9. doi: https://doi.org/10.1016/j.aninu.2017.01.001.
2. Klem TB, Bleken E, Morberg H, Thoresen SI, Framstad T. Hematologic and biochemical reference intervals for Norwegian crossbreed grower pigs. Veterinary Clinical Pathology. 2010;39(2):221-6. doi: https://doi.org/10.1111/j.1939-165X.2009.00199.x.
